# Advancing Positron Imaging with Alginate-Based Tracers: Design, Synthesis, and Radiolabelling Methods^☆^

**DOI:** 10.1016/j.mex.2026.103927

**Published:** 2026-04-22

**Authors:** Chloe Huckvale Bruno, Daniele Baiocco, William J. Peace, Dawid Hampel, Manikandan Kadirvel, Ben Phoenix, Emma Catterson, Zhibing Zhang, Carl Wheldon, Christopher Windows-Yule

**Affiliations:** a School of Physics and Astronomy, University of Birmingham, Edgbaston, Birmingham, B15 2TT, The United Kingdom; b School of Chemical Engineering, University of Birmingham, Edgbaston, Birmingham, B15 2TT, The United Kingdom; c School of Chemistry, University of Birmingham, Edgbaston, Birmingham, B15 2TT, The United Kingdom; d The Institute for Data and AI (IDAI), University of Birmingham, Edgbaston, Birmingham, B15 2TT, The United Kingdom; e Department of Chemical Engineering, School of Aeronautical, Automotive, Chemical and Materials Engineering, Loughborough University, Epinal Way, Loughborough, LE11 3TU

**Keywords:** Particle tracking, Nuclear imaging, Alginate, Fluoride adsorption

## Abstract

Nuclear imaging plays a vital role in both medical diagnostics and the flow visualisation of engineering systems. Among these techniques, Positron Emission Particle Tracking (PEPT) and Positron Emission Tomography (PET) are renowned for their ability to provide high-resolution tracking of radioactive materials in industrial and biological environments. The success of these methods relies on the effective radiolabelling of materials with suitable radioisotopes, ensuring high activation efficiency and optimal tracer properties. However, the radiolabelling of solid particulates, which is essential for PEPT and increasingly relevant for PET, remains technically challenging. To overcome these limitations, this work presents a methods for the formulation, fabrication, and radiolabelling of “designer” tracer particles, utilizing a porous alginate-based hydrogel matrix combined with an evaporative radiolabelling technique. This approach enables the production of small, mechanically robust tracers exhibiting high single-particle activity and radiochemical stability. This document details the synthesis, characterisation, and radiolabelling methodologies developed to produce these tracers, along with a simple extrusion dripping process that allows customisation of tracer properties through controlled formulation and operating parameters. The key components of this method are:

• The synthesis of alginate hydrogels through extrusion dripping.

• The characterisation of tracer particles.

• The radio-labelling procedure to obtain particle tracers.

## Graphical abstract

**Figure:**
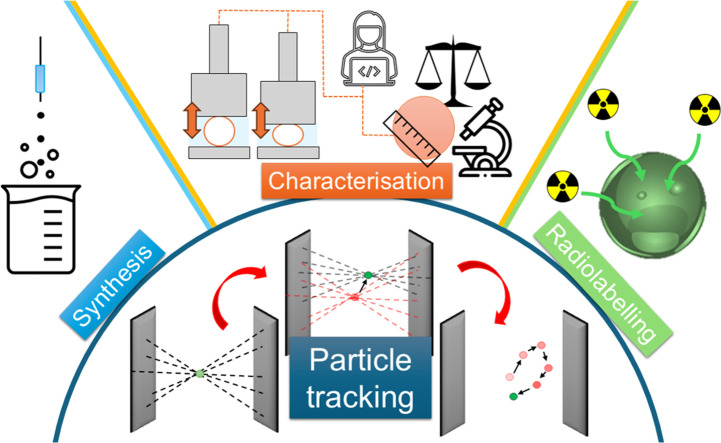

**Specifications table**

**Table:** 

**Subject area**	Physics and Astronomy
**More specific subject area**	Nuclear Imaging
**Name of your method**	Preparation and radiolabelling of alginate-based tracers
**Name and reference of original method**	N/A
**Resource availability**	N/A

## Background

Positron Emission Particle Tracking (PEPT) is an advanced imaging technique based on the principles of nuclear imaging. It involves the use of one or more radioactive tracer particles to probe the fluid and/or granular particle dynamics occurring inside optically opaque industrial and/or biological systems [1]. Originating from the principles of Positron Emission Tomography (PET), PEPT shares several conceptual similarities with this medical imaging technique. However, it diverges in two key aspects: the use of a discrete radioactive tracer particle rather than a distributed tracer fluid, and its primary application in optimizing industrial processes and unit operations rather than medical diagnostics [2].

In brief, a suitable tracer particle is labelled with an appropriate radionuclide through either direct or indirect activation methods. The labelled particle is then transferred to the desired equipment to be examined, where the equipment is situated between a dual-head positron camera. The tracer follows the fluid streamlines or granular particle trajectories which occur inside the equipment during its operation for liquid and granular systems respectively, whilst simultaneously undergoing radioactive decay [3,4]. The radionuclide attached to or embedded within the tracer ideally undergoes beta-decay with a high positron decay ratio and low energy to preserve both the imaging quality and resolution [5]. This involves the production and subsequent annihilation of a positron, resulting in the emission of two gamma photons with back-to-back trajectories. These photons are detected by the positron cameras, whereby various triangulation algorithms subsequently allow the geometric centroid of the single discrete radiolabelled particle to be directly evaluated from multiple lines of response with high precision given multiple annihilation events and a short positron diffusion range [2]. This results in the output of data containing the particle location at various time-steps throughout the equipment operation, thus allowing quantification of a vast array of information about the dynamics of a system [6]. This type of high-resolution spatio-temporal data has been used to allow both optimisation of unit operations and validation of computational dynamic simulations, and thus is an invaluable tool for the imaging of optically opaque industrial systems [7]. A future aspiration is thus to extend PEPT towards clinical applications, where such high-resolution particle tracking could enable novel prognostic and diagnostic approaches.

**Fig. 1:**
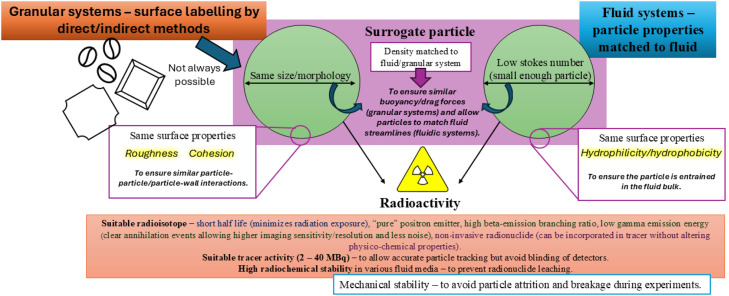
Requirements for an ideal PEPT tracer.

A key component of this experimental technique is the production of a sufficiently active, chemically and physically stable tracer particle with suitable physical and chemical characteristics designed to match the qualities of the imaging media. In practice, this aspect is non-trivial, though it is often an overlooked and under-reported aspect of the PEPT technique. Fig. 1 visualises the key characteristics and specifications of tracer particles destined for use in PEPT experiments, highlighting the requirements of:

• Appropriate decay and radiochemical properties to allow high imaging resolution and ensure the accuracy of the PEPT technique.
• Matched physical and surface properties with regards to the system of interest, ensuring the particle acts as a true "non-invasive" tracer.
• High mechanical robustness to ensure the tracer can withstand the high-shear environments which may be present within industrial equipment.

Considering the wide variety of potential applications of PEPT [3,[6], [7], [8], [9]], with liquid and granular media densities varying from 1 - 4 g/cm^3^ [10,11] and granular particle sizes ranging from 0.95 *μ*m - 5 cm [[12], [13], [14]], among other variations, the importance of procuring, characterising, and effectively radiolabelling a vast array of particles with differing properties becomes evident.

This article will thus detail the methods which can be used to easily synthesise, efficiently radio-label, and accurately characterise customisable alginate-based tracer particles with the fluorine-18 (^18^F) radionuclide – the most commonly used radioisotope for PEPT and PET applications due to its favourable decay characteristics [[15], [16], [17]]. The alginate particle formulation can be varied to obtain different properties suitable for multiple applications, and typically provides extremely robust tracers with favourable radiochemical properties, as has been detailed in the sister publication to this work [18]. The methodological novelty of this work lies in the ability to systematically tailor key tracer properties through minor adjustments to formulation and preparation conditions. This is enabled by the fabrication of a porous alginate hydrogel with high fluoride affinity through simple extrusion dripping, which can be either left in its hydrogel form or converted to a xerogel to obtain more robust radiochemical and mechanical properties through the use of a low temperature or evaporative adsorption method respectively. These methods thus provide control over tracer robustness, radiolabelling efficiency, and preparation flexibility, offering a versatile and reproducible platform for tracer development and future optimization for more bespoke applications. Furthermore, this work provides the first in-depth description of PEPT tracer preparation, from the synthesis of bespoke particles themselves to their various radiolabelling methods.

## Method details

### Generalised workflow for the preparation and radiolabelling of alginate-based tracer particles

**Fig. 2:**
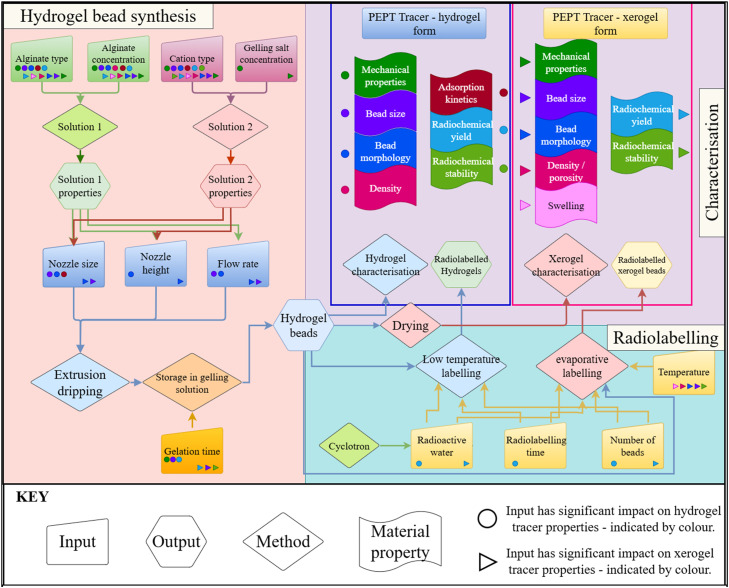
General workflow of methods.

Fig. 2 displays an overview of the complete, method for synthesising, radiolabelling, and characterising alginate‑based tracer particles, with the general workflow steps including:

1. Synthesis of alginate-based hydrogel beads
  ➢ Preparation of solutions
  ➢ Extrusion dripping and subsequent gelling
2. Use of hydrogel beads for:
  ➢ Hydrogel characterisation
  ➢ Drying and subsequent xerogel characterisation
  ➢ Radiolabelling via either
    Low temperature radiolabelling to obtain wet hydrogel tracers.
    Evaporative labelling to obtain dry xerogel tracers.

Each stage, with clear definitions of the required inputs, operational parameters, decision rules, and expected outputs is detailed below.

### Synthesis of alginate-based hydrogel beads

**Fig. 3:**
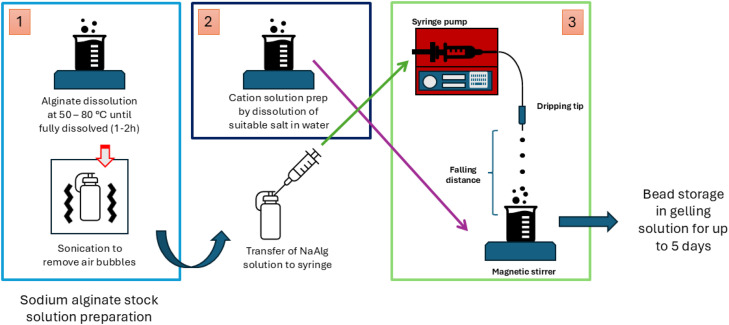
Synthesis of alginate hydrogel beads through extrusion dripping: 1. Preparation of solution 1 (aqueous alginate salt solution), 2. Preparation of solution 2 (aqueous chloride salt solution), 3. Extrusion dripping with correct parameter selection.

The synthesis of alginate-based hydrogel beads involves several steps, visualised in Fig. 2, Fig. 3, where Fig. 3 provides a visual aid for the synthesis technique whilst Fig. 2 emphasises the key inputs, outputs and relations of the synthesis step to tracer labelling and bead properties. The details of these visualizations are discussed below.

#### Preparation of solution 1

Two separate solutions must be prepared, the first being solution 1, as it requires several hours to fully mix and dissolve the components. Solution 1 consists of a sodium alginate (NaAlg) polymer salt of the required specifications dissolved in distilled water. The key input parameters are:

• **The NaAlg type**, specifically the mannuronic-to-guluronic acid monomer ratio (MGR).
  **Output effect on solution properties**
    ▪ **Viscosity:** higher values provide less viscous solutions.
  **Output effect on final bead properties**
    ▪ **Gel structure:** higher values provide weaker gels of *lower crosslinking density* and thus;
      ➢ lower mechanical strength,
      ➢ lower density,
      ➢ larger bead size (less syneresis upon gelation - larger $k_{SF}$ in Eq. (1) below),
      ➢ higher porosity in dried form,
      ➢ greater swelling ability, and
      ➢ less cationic sites available for radionuclide adsorption – less favourable radiolabelling properties.
  **Typical range:** MGRs of 0.5 - 1.6 are typically available from commercial suppliers.
• **The NaAlg concentration**
  **Output effect on solution properties**
    ▪ **Viscosity:** higher values provide more viscous solutions.
  **Output effect on final bead properties**
    ▪ **Gel structure:** higher values provide stronger gels of *higher crosslinking density* and thus;
      ➢ greater mechanical strength,
      ➢ higher density,
      ➢ lower porosity in dried form,
      ➢ less swelling ability, and
      ➢ more cationic sites available for radionuclide adsorption – more favourable radiolabelling properties.
  **Suggested range** for mechanically stable beads with manageable viscosity:
    ▪ Low MGR: between 0.5 - 4 wt.%.
    ▪ High MGR: between 2 – 6 wt.%

The method to obtain solution 1 is as follows:

1. The appropriate amount of sodium alginate salt in powder form and corresponding amount of distilled water are separately weighed out using any suitable scale with a precision of at least 0.01 g to achieve the desired concentration of NaAlg.
2. The powder is then slowly added to a beaker containing the distilled water whilst being agitated on a hot plate equipped with a magnetic stirrer. The solution must be left to dissolve for 1 - 2 h at a temperature of typically 50–80 °C (time-temperature combination for dissolution should be decided based on recommendations from the NaAlg supplier),
3. The solution is then sonicated to remove entrained air bubbles. Sonication is required as air bubbles may be difficult to remove by relying solely on buoyancy forces due to the high viscosity of some solutions (depending on the alginate type and NaAlg concentration), and should be applied using typical conditions of 20–50 kHz for 10–30 min, or until the solution appears clear of visual air bubbles.
• The key output parameters and impacts associated with solution 1 are: **Solution 1 viscosity and surface tension**, with direct effect on the extrusion dripping parameters selection to achieve given bead properties, where higher values typically cause:
  Greater minimum achievable bead size by extrusion dripping.
  Higher likelihood to obtain non-spherical beads.
  Higher likelihood of extrusion dripping nozzle failure due to extreme pressure drop.

#### Preparation of solution 2

Solution 2 consists of an inorganic chloride salt containing the desired metal cation to instigate alginate gelation, dissolved in distilled water. The key input parameters are:

• **The metal salt concentration -** the concentration must be high enough to ensure there is an abundance of metallic cations available for alginate gelation (i.e. enough to completely saturate the alginate carboxyl groups and thus maximise cross-linking [[19], [20], [21]]).
  **Suggested ranges:** The weight-based concentration should be at least double that of NaAlg in solution 1, or no <0.1 M [19].
• **The gelling metal cation type**
  **Output effect on solution properties**
    ▪ **Surface tension**: certain chloride salts may increase the solution surface tension.
  **Output effect on final bead properties**
    ▪ **Gel structure -** the cation valence and ionic potential will affect both the crosslinking density and structure, with varying direct impacts on:
      ➢ the bead size ($k_{SF}$ in Eq. (1) below) and morphology,
      ➢ dry bead density and porosity,
      ➢ dry bead swelling, and
      ➢ mechanical properties,
    ▪ **Fluoride affinity -** the cation valence and ionic potential will affect the fluoride affinity of the bead, with varying direct impacts on:
      ➢ the radiochemical yield, and
      ➢ the radiochemical stability.
  **Suggested values:**
    ▪ **Multivalent cations** are required to allow alginate gelation, thus the cation valence in solution must be at least ≥ +2. Preferably the valence is ≥ +3, with small ionic size, as this provides gels of higher charge density and thus fluoride affinity.
      ➢ At low MGR, the bead physical properties of trivalent cation-alginate gels are much less sensitive to the cation type.
      ➢ At high MGR, certain cation types (e.g. Al^3+^, Fe^3+^, and Ce^3+^) have been found to provide highly irregular and porous beads with less favourable physical properties, hence care should be taken when formulating with these types of NaAlg.

The method to obtain solution 2 is as follows:

1. The appropriate amount of inorganic chloride salt in crystalline form and corresponding amount of distilled water are separately weighed out using any suitable scale with a precision of at least 0.01 g to achieve the desired salt concentration.
• If the salt is in hydrous form, the hydration in the crystals must be accounted for when considering the mass required.
2. The salt crystals are then added to the distilled water in a beaker at room temperature whilst stirring using a magnetic stirrer.
3. Stirring is continued until the salt crystals are dissolved (5 – 10 min).

The key output parameters and impacts associated with solution 2 are:

• **Solution 2 surface tension**, with a small effect on the accuracy of the preliminary design calculations for parameter selection in the extrusion dripping process described below. Specifically, since the design Eqs. (4)–5 are empirical, and were based on extrusion dripping into calcium chloride solutions, a solution 2 formulation with different cation type may provide varying results depending on the extent of deviation of the surface tension. Higher surface tension values typically cause:
  Higher likelihood to obtain flattened disk-shaped beads.
  Lower fidelity of the maximum falling height within which a spherical droplet will be obtained (Eq. (5) below).

#### Extrusion dripping

**Fig. 4:**
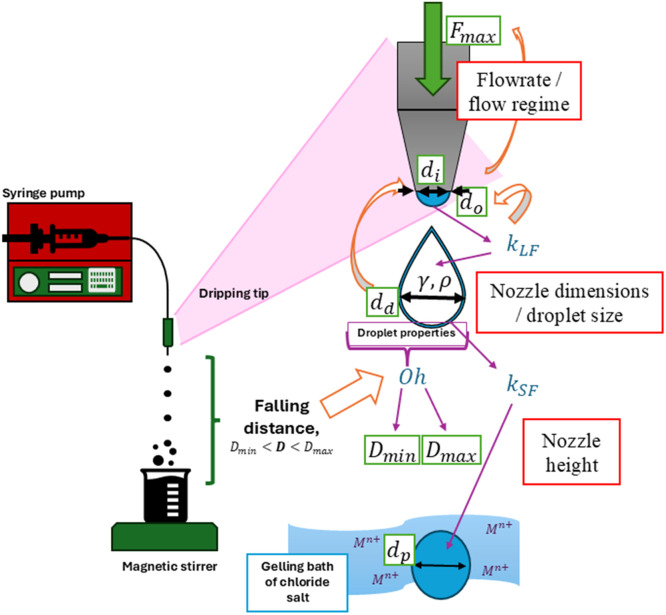
Set-up and key parameters for production of alginate hydrogels via extrusion dripping.

Solutions 1 and 2 can then be combined in a specific manner to enable alginate hydrogel beads to be formed. This is done through the technique known as extrusion dripping (ExD). The ExD process and equipment components are depicted in Fig. 4. This Figure highlights the key parameters which must be considered to enable the production of homogenous, spherical, and monodisperse hydrogel beads.

The ExD set-up design must ensure droplets of the desired shape and size are obtained, and hence requires careful consideration and selection of input parameters. The key input parameters are:

• **Solution 1 viscosity and surface tension,** as dictated by the solution 1 preparation method specified above.
  **Output effect on final bead properties**
    ▪ **Bead size:** Determines the minimum achievable bead size by extrusion dripping.
    ▪ **Bead shape:** Can change the final bead shape due to variation in the Ohnesorge number (Eq. (6)).
• **Solution 2 surface tension,** as dictated by the solution 2 preparation method specified above.
  **Output effect on final bead properties**
    ▪ **Bead shape:** higher values typically cause higher likelihood to obtain flattened beads.
• **The required nozzle diameter to achieve a given bead size.**
  **Output effect on final bead properties**
    ▪ **Bead shape and size:** directly affects the bead size, and thus can change the final bead shape due to variation in the Ohnesorge number (Eq. (6)).
  **Suggested ranges –** the correct nozzle size to achieve a specific droplet diameter is determined from Eqs. (1)–2 in the design calculations presented below.
    ▪ Caution is advised when operating at high viscosity (high solution 1 concentration and/or lower MGR), as the pressure drop may become excessive and cause nozzle failure.
• **Solution 1 flow rate through the nozzle** - to ensure the fluid is in the dripping regime upon exiting the nozzle tip.
  **Output effect on final bead properties**
    ▪ **Size distribution:** flow rates above the critical jetting velocity will cause irregular and polydisperse beads to be obtained, whereas operation at low flow rate enables the production of monodisperse and uniform beads.
    ▪ **Bead size:** droplet size typically increases at high flow rates within the stable dripping regime [19].
  **Suggested ranges –** the maximum allowable flowrate to avoid fluid jetting is determined from the design calculations presented below (Eq. (3)).
    ▪ In practice, the Eq. (3) denotes the maximum flow rate upper boundary, and a much lower value should be applied to ensure the pump pressure is not excessive and a stable dripping regime that gives reproducible, spherical beads is maintained. This is especially important if a small droplet size is desired, hence the selection of flow rate must be balanced with the desired throughput.
• **The optimal falling distance window** within which a spherical droplet will be obtained.
  **Output effect on final bead properties**
    ▪ **Bead shape:**
      ➢ Extremely small falling distances outside the optimal spherical range will cause misshapen droplet-type beads to be formed.
      ➢ Extremely large falling distances outside the optimal spherical range will cause misshapen cylindrical or flattened beads to be formed.
    ▪ **Mechanical properties**: non-spherical beads may alter the mechanical behaviour of the final tracers, as misshapen beads can be more prone to attrition and/or bead rupture.
  **Suggested ranges –** the chosen falling distance to achieve a spherical droplet shape should be within the limits denoted by Eqs. (4)–5. If non-spherical droplets are desired, operation should occur outside these limits. Further details regarding possible droplet shapes are described by Chan et al. (2009) [22].
• **Ohnesorge number -** the ratio of viscous to surface tension forces in the liquid drop (Eq. (6) below).
  **Output effect on final bead properties**
    ▪ **Bead shape**: an Oh number that is too low indicates an insufficient droplet viscosity which cannot resist the forces of deformation upon impact with the gelling solution surface, whereas excessive viscosity and low surface tension will tend to preserve the shape of the detached droplet.
    ▪ **Mechanical properties**: non-spherical beads may alter the mechanical behaviour of the final tracers, as misshapen beads can be more prone to attrition and/or bead rupture.
  **Suggested ranges –** for spherical droplet formation, the Oh number must be between 0.24 and 11, though if non-spherical droplets are desired, operation could occur outside these limits. Further details regarding possible droplet shapes are described by Chan et al. (2009) [22].
• **Gelling time –** hydrogel beads formed via extrusion dripping are left to mature in the gelling solution under refrigerated conditions for a given gelling time.
  **Output effect on final bead properties –** Longer gelling time ensures cross-linking reaches completion, with impacts on:
    ▪ **Mechanical properties**: bead strength and stiffness improve until maximum crosslinking is achieved.
    ▪ **Bead size**: shorter gelation times may cause incomplete crosslinking, and thus larger bead size (less syneresis - larger $k_{SF}$ in Eq. (1) below).
  **Suggested ranges –** at least 24 h should be allowed to achieve full gelation, though more conservative times of up to five days under refrigerated conditions (4 °C) are recommended for beads of novel formulation with large size and high NaAlg concentration in solution 1, as both factors are expected to exacerbate mass-transfer limitations.

### Preliminary design calculations for extrusion dripping

The parameters in Fig. 4 are selected following guidance from design equations originally outlined by Lindblad and Schneider (1965) [23] and later elaborated by Chan et al. (2009) [22]. To aid reader comprehension, an example design calculation has been detailed in the supplementary materials for a specific case.

#### Nozzle diameter

The final bead size is predicted from an equation based on Tate’s law, which has been modified by Chan et al. (2009) [22]:

(1) $$d_{p}=k_{LF}k_{SF}{\left(6\frac{d_{o}\gamma }{\rho g}\right)}^{\frac{1}{3}}=K{\left(6\frac{d_{o}\gamma }{\rho g}\right)}^{\frac{1}{3}}.$$

where, $d_{p}$, is the final bead diameter, *K* - which combines the liquid loss factor, $k_{LF}$, to account for liquid lost at the nozzle tip upon detachment [24], as well as the shrinkage factor, $k_{SF}$, to account for bead shrinkage upon gelation - is an empirical constant, $d_{o}$ is the outer nozzle diameter, γ is the surface tension of solution 1, ρ is the density of solution 1, and *g* is the gravitational constant. These constants are determined by considering both the selected nozzle and the alginate type. In particular, $k_{LF}$, has been directly related to the nozzle size:

(2) $$k_{LF}=0.98-0.04d_{o};$$

where $d_{o}$ is expressed in millimetres.

The shrinkage factor is more complex to establish. The value has been found to range from 0.5 - 0.9 for varying conditions [19], and strongly depends on the type of alginate employed [22,25,26] as well as the gelling conditions, including the gelling cation from solution 2, and gelling time [27], hence it typically must be determined empirically [22]. The consideration of gelling cation is of particular importance, since variations from the typically employed calcium cation significantly alters the alginate gel structure and porosity, and thus extent of syneresis, especially when employing alginate of high MGR. Nevertheless, conservative estimates based on previous findings for given ExD configurations and specific formulations can allow for further design calculations to be made.

Thus, Eqs. (1)–2 can be solved iteratively or using a numerical solver to determine the nozzle diameter required to achieve a given hydrogel bead diameter.

#### Solution 1 flow rate

Once the nozzle dimensions are selected based on the desired droplet size, the maximum allowable flow rate through the nozzle must be determined to ensure operation occurs within the dripping regime. The maximum drop-wise flow rate, *F_max_*, above which fluid jetting commences, is calculated from:

(3) $$F_{max}=\frac{\pi d_{i}^{2}}{2}\sqrt{\frac{\gamma }{\rho d_{i}}},$$

where *d_i_* is the selected nozzle internal diameter [28]. In practice, this denotes the maximum flow rate upper boundary, and a much lower flow rate should be applied to ensure the pump pressure is not excessive and a stable dripping regime that gives reproducible, spherical beads is maintained. This is especially important if a small droplet size is desired, since the droplet size typically increases at high flow rates [19], hence the selection of flow rate must be balanced with the desired throughput.

#### Optimal spherical bead falling distance and Ohnesorge number

The minimum and maximum collecting distances, $D_{min}$ and $D_{max}$ respectively, within which spherical droplets can be obtained, have been described by Chan et al. (2009) [22]:

(4) $$D_{min}=1.63e^{0.12\text{Oh}},$$

(5) $$D_{max}=62.35\ln\text{Oh}+111,$$

where Oh refers to the Ohnesorge number. The Oh number describes the ratio of viscous to surface tension forces in the liquid drop, and is given by:

(6) $$\text{Oh}=\frac{\mu }{\sqrt{\rho \frac{d_{p}}{k_{SF}}\gamma }}=\frac{\mu }{\sqrt{\rho d_{d}\gamma }},$$

where *d_d_* is the droplet diameter before gelation, and μ is the dynamic viscosity of solution 1 [19]. This group is also critical to consider in general for spherical droplet formation, since an Oh number that is too low (typically < 0.24) indicates an insufficient droplet viscosity which cannot resist the forces of deformation upon impact with the gelling solution surface, whereas excessive viscosity and low surface tension (or Oh > 11) will tend to preserve the shape of the detached droplet – thus, for constant droplet size and alginate type, Eq. (6) can also allow the minimum and maximum allowable alginate concentration to be determined, since the concentration will directly impact the surface tension and solution viscosity. The final shape will depend on both Oh and the chosen falling distance from the nozzle to the gelling solution surface, which is accounted for using the guidelines set out by Eqs. (4) - 5 [[19], [22],^28^].

#### Preliminary design calculations summary and limitations

The above calculations, with their inputs, outputs, and calculation method, are summarized in Table 1.

**Table 1:** Design calculations and guidelines summary.

Independent variable	Equation inputs / dependent variables	Input type	Typical ranges and dependencies	Equation format	Calculation method
*Nozzle external diameter,*$d_{o}$	Desired final gelled particle diameter, $d_{p}$	Key parameter / dependent variable	1 – 5 mm, selected by user	$d_{p}=k_{LF}k_{SF}{\left(6\frac{d_{o}\gamma }{\rho g}\right)}^{\frac{1}{3}}$; $k_{LF}=0.98-0.04d_{o};$	Given solution 1 properties, estimate the shrinkage factor. Decide the desired final gelled droplet diameter and thus use an iterative procedure or numerical solver to calculate required nozzle size.
	Loss factor, $k_{LF}$	Empirical value, determined from $d_{o}$	For nozzle sizes from 0.16 – 3.4 mm, $k_{LF}$ is 0.97–0.84.		
	Shrinkage factor, $k_{SF}$	Empirical constant	Varies from 0.5–0.8, and depends on the formulation and gelling conditions.		
	Surface tension, γ	Property of solution 1	Varies slightly with NaAlg concentration in solution 1, from 0.071 – 0.047 N/m for concentrations of 0.5 – 5 [22]		
	Solution density, ρ	Property of solution 1	Varies slightly with NaAlg concentration in solution 1, from 999 – 1023 N/m for concentrations of 0.5 – 5 [22]		
	Gravitational constant, g	Physical constant	9.81		
*Maximum allowable flow-rate,*$F_{max}$	Internal nozzle diameter, $d_{i}$	Key parameter / dependent variable	Corresponds to the external nozzle diameter, $d_{o}$, as calculated from the above. Typically 0.051 – 2.7 mm.	$F_{max}=\frac{\pi d_{i}^{2}}{2}\sqrt{\frac{\gamma }{\rho d_{i}}}$	Given solution 1 properties, and required nozzle dimensions – as calculated from the above – determine the maximum allowable flow-rate to remain within the dripping regime.
	Surface tension, γ	Property of solution 1	Varies slightly with NaAlg concentration in solution 1, from 0.071 – 0.047 N/m for concentrations of 0.5 – 5 [22].		
	Solution density, ρ	Property of solution 1	Varies slightly with NaAlg concentration in solution 1, from 999 – 1023 kg/m^3^ for concentrations of 0.5 – 5 [22].		
*Ohnesorge number*	Pre-gelled droplet diameter, $d_{d}$	Key parameter / dependent variable	Determined from the relationship $d_{p}=k_{SF}d_{d}$, typically 1.1 – 10 mm.	$\text{Oh}=\frac{\mu }{\sqrt{\rho d_{d}\gamma }}$	Given solution 1 properties and desired droplet diameter, determine the Oh number to enable further design calculations.
	Dynamic viscosity, μ	Property of solution 1	Varies slightly with NaAlg concentration and MGR in solution 1, from 0.03 – 5 Pa s for concentrations of 0.5 – 5 [22].		
	Surface tension, γ	Property of solution 1	Varies slightly with NaAlg concentration in solution 1, can range from 0.071 – 0.047 N/m for concentrations of 0.5 – 5 [22].		
*Minimum collecting distance,*$D_{min}$	Ohnesorge number, Oh	Key parameter / dependent variable	Calculated from the above. Must be > 0.24 and < 11 for spherical beads to be obtained.	$D_{min}=1.63e^{0.12\text{Oh}}$	Given the Oh number, calculated from the above, determine the minimum collecting distance required to obtain spherical droplets.
*Maximum collecting distance,*$D_{max}$	Ohnesorge number, Oh	Key parameter / dependent variable	Calculated from the above. Must be > 0.24 and < 11 for spherical beads to be obtained.	$D_{max}=62.35\ln\text{Oh}+111$	Given the Oh number, calculated from the above, determine the maximum collecting distance required to obtain spherical droplets.

It is critical to note that the design Eqs. (1)–6 were derived for extrusion dripping of alginate into calcium chloride solutions. Substituting other chloride salts as gelling agents can modify key correlation parameters. Stronger or weaker interactions between alginate and the gelling cation, as well as variations in the cross-linking structure, may influence the degree of droplet shrinkage (reflected in lower or higher $k_{SF}$), thereby altering the relationship between the final bead diameter and the nozzle dimensions. In addition, a higher surface tension of solution 2 could shift the required falling distance for spherical droplet formation, as increased impact forces may flatten the bead at excessive surface tensions. For these reasons, the design equations should be regarded as preliminary guidelines rather than absolute rules.

### Extrusion dripping method

Once the nozzle, flow rate, and collection distance have been appropriately selected, the ExD procedure for producing uniform alginate hydrogel beads can be carried out through the following method:

1. Load solution 1 into a syringe equipped with a luer-lock tip. Attach the syringe to a PTFE tube connected to the extrusion dripping nozzle (see Fig. 3). Seal the nozzle fittings with parafilm to minimize leakage of solution 1.
2. Place the beaker containing solution 2 on a magnetic stirrer beneath a support stand with a clamp.
3. Secure the ExD nozzle in the clamp such that the nozzle tip is approximately positioned at the desired height above the surface of solution 2. Fix the syringe body into a syringe pump adjacent to the stand.
4. Temporarily remove the beaker containing solution 2 from beneath the nozzle. Start the syringe pump at a slightly higher flow rate than that selected above, and allow fluid to run through the nozzle for 30 - 60 s to flush out any impurities in the tubing. Note the deposition area on the stirrer plate.
5. Lower the flow rate to the selected value. Return the beaker of solution 2 beneath the nozzle, positioning it so that droplets fall slightly off-centre from the m50le of the beaker, away from the stirrer vortex. Maintain stirring at approximately 500–550 rpm.
6. Continue extrusion, allowing droplets to fall into solution 2 until the desired quantity of beads is obtained or the syringe is emptied.
7. The resultant hydrogel beads are left to mature in the gelling solution under refrigerated conditions for the desired gelation time.
8. Subsequently, the beads are separated from the gelling solution using a mesh screen of appropriate size, and thoroughly rinsed with distilled water.
9. The washed beads are stored in distilled water under refrigerated conditions until further use.

### Extrusion dripping output

The key output parameters and impacts associated with the hydrogel beads formed from extrusion dripping are:

• **Hydrogel beads of given formulation** - determined by the inputs to solutions 1 and 2 - **and size/morphology** – influences by both the formulation and extrusion dripping parameters – with corresponding physical and radiochemical properties, ready for use in:
  Radiolabelling procedures (low temperature or evaporative adsorption)
  Hydrogel characterisation methods.
  Bead drying to obtain xerogels for dry bead characterisation methods.

Radiolabelling of Alginate-Based Hydrogel Beads

The obtained hydrogels are both small and sensitive to temperature, hence they are better suited to labelling using indirect rather than direct techniques [6]. Two approaches of varying efficacy are described herein, both relying on the adsorption of a suitable radionuclide from an aqueous solution, but ultimately delivering different final tracer properties;

1. Low-temperature – maintains hydrogels in their hydrated state.
2. Evaporative adsorption – dehydrates hydrogel beads whilst simultaneously adsorbing the radionuclide.

A comparison of the tracer properties obtained using the two different methods is given in Table 2, which can be used as an aid to inform which method is appropriate for a given application.

**Table 2:** Comparison of the different properties achieved for the evaporative and low-temperature radiolabelling methods.

Parameter	Low temperature labelling	Evaporative labelling
*Tracer type*	Hydrated hydrogel bead	Dry xerogel particle
*Tracer size range (equivalent diameter, mm)*	1 – 10	0.4 – 4
*Tracer true density (kg/m^3^)*	1 – 1.2	1.4 – 2.3
*Tracer mechanical properties*	Soft and flexible/deformable beads with high rupture strain (> 50%), and low Young’s modulus (order of MPa)	Hard and rigid particles with low rupture strain (< 10%), and high Young’s modulus/yield stress (order of GPa)
*Tracer morphology*	As determined by extrusion dripping parameters, where it is easy to achieve highly uniform and spherical beads.	Sphericity is diminished upon labelling due to the uncontrolled nature of the drying process.
*Tracer surface properties*	Hydrophilic / lipophobic	Amphiphilic
*Tracer radiochemical yield*	Adsorption is typically governed by Henry’s law due to extremely low radionuclide concentrations, thus the RCY is typically always relatively lower for a given tracer formulation.	Adsorption is decoupled from initial concentration due to the evaporative process, thus the RCY is typically always relatively higher for a given tracer formulation.
*Tracer radiochemical stability*	Adsorption at low temperature can typically involve both chemisorption and physisorption, whilst the porous hydrogel form is maintained, resulting in relatively lower RCS for a given tracer formulation.	Adsorption at high temperature allows for more chemisorption-based mechanisms to occur, whilst the porous hydrogel form is collapsed into a denser xerogel form at the end of the labelling, potentially providing steric hinderance, resulting in a much higher RCS for a given tracer formulation.
*Suggested applications*	Aqueous fluid-based systems requiring density close to water, where larger tracers with higher levels of radioactive leaching can be tolerated.	Fluid or granular systems requiring small tracers and/or high densities, high mechanical resistance, high tracer activity, and low levels of radioactive leaching.

Preliminary calculations for the radiolabelling of radioactive materials

For any radioactive material, the volumetric activity density, $\rho_{A}$, is defined as

(7) $$\rho_{A}=\frac{A}{V};$$

where *A* is the activity measured at a given time, and *V* is the volume of the material. The molar concentration of active radioisotopes, *C*, can be determined from:

(8) $$C=\frac{A}{\lambda N_{A}V}=\frac{\rho_{A}}{\lambda N_{A}};$$

where *λ* is the decay constant for ^18^F, and $N_{A}$ is Avogadro’s constant [29]. The temporal variation in the material activity is accounted for using the basic equation for radioactive decay [30];

(9) $$A=A_{0}e^{-\lambda t};$$

where $A_{0}$ is the initial activity at reference time *t* = 0. Thus, in any indirect tracer radiolabelling process in which a radionuclide is transferred via adsorption from a solution to a given number of adsorbent beads (tracers) in a single radiolabelling vessel, the final activity of any individual tracer, $A_{b}$, labelled in such a manner can be estimated from:

(10) $$A_{b}=\text{RCY}\;\times \frac{A_{T0}e^{-\lambda t_{r}}}{n}=\text{RCY}\;\times \frac{\rho_{A,T0}V_{T}e^{-\lambda t_{r}}}{n},$$

where RCY is the average radiochemical yield of this process, considering both the process parameters and tracer type, $A_{T0}$ is the initial activity associated with the total volume of radioactive solution, $V_{T}$, introduced to the radiolabelling vessel of initial volumetric activity density denoted by $\rho_{A,T0}$, n is the number of beads undergoing radiolabelling in the same vessel, and $t_{r}$ is the radiolabelling time.

Obtaining the Aqueous Radionuclide Solution

Both radiolabelling methods described herein require radioactive water containing the ^18^F radionuclide in the form of solvated anions. The water is produced using a low energy medical cyclotron capable of accelerating protons up to 12 MeV (an MC40 Cyclotron at the University of Birmingham [31] was used by the present research group). A proton beam is directed at a water target enriched in the ^18^O isotope to induce the ^18^O(*p,n*) ^18^F reaction [32], where the key inputs for this procedure include:

• **The level of ^18^O isotope enrichment in the water target**
  **Output effect on final tracer properties**
    ▪ **Tracer activity:** higher levels enhance the final water activity density, and thus allow more active tracers to be obtained.
      ➢ Less active tracers can still be produced from high activity density water through dilution with distilled water, thus optimal values to achieve higher activity density are typically always more desirable.
    ▪ **Radionuclide purity:** higher ^18^O enrichment suppresses competing nuclear reactions on ^16^O, thereby reducing the formation of unwanted radionuclides. Lower enrichment increases the proportion of unwanted radionuclides, which can compromise tracer performance.
  **Suggested ranges:** The level of enrichment can be varied depending on the desired final activity, whilst considering the economic cost of the enriched water, and can typically range anywhere from 0.7 – 100% enrichment.
    ▪ For example, the present research team commonly employs water enriched to 6.25%.
• **The proton beam energy** - tailored to achieve a high probability (nuclear cross-section) of the desired nuclear pathway, and low probability of side reactions.
  **Output effect on final tracer properties**
    ▪ **Tracer activity:** optimum beam energy increases the nuclear cross-section and thus the yield of ^18^F, providing greater final water activity density, and thus allowing more active tracers to be obtained.
      ➢ Less active tracers can still be produced from high activity density water through dilution with distilled water, thus optimal values to achieve higher activity density are typically always more desirable.
    ▪ **Radiochemical purity:** Sub‑optimal beam energy can promote competing nuclear reactions, leading to the formation of unwanted radionuclides that reduce the purity of the product and compromise tracer performance.
  **Suggested ranges:** the ^18^O(*p,n*) ^18^F reaction has a threshold energy of > 2.6 MeV, and an optimal range between 10 – 15 MeV.
• **The proton beam current** - determines the number of protons impacting the target per second.
  **Output effect on final tracer properties**
    ▪ **Tracer activity:** directly affects the radionuclide yield, thus higher levels enhance the final water activity density and allow more active tracers to be obtained.
      ➢ Less active tracers can still be produced from high activity density water through dilution with distilled water, thus optimal values to achieve higher activity density are typically always more desirable.
    ▪ **Radiochemical purity:** Higher beam currents increase the rate of side reactions and can enhance radiolysis within the target water. This may generate chemical impurities or promote the formation of unwanted radionuclides, ultimately reducing the radiochemical purity of the final tracer and compromising its performance.
  **Suggested ranges:** The current must be sufficient to achieve the desired activity density and is typically between 10 – 60 *μ*A.
    ▪ Excessive current can cause overheating, target damage, and radiolysis, and thus must be tailored to the specific target design.
• **The irradiation time** - influences the accumulated activity until saturation is approached due to radioactive decay.
  **Output effect on final tracer properties**
    ▪ **Tracer activity:** increases the accumulated activity until saturation is approached due to radioactive decay, thus the optimal time will provide higher final water activity density and allow more active tracers to be obtained.
      ➢ Less active tracers can still be produced from high activity density water through dilution with distilled water, thus optimal values to achieve higher activity density are typically always more desirable.
  **Suggested ranges:** for the ^18^F radionuclide, saturation is approached after approximately 5.5 h. Typical irradiation time should thus be between 1 – 2 h due to diminishing returns.
    ▪ Further increases in yield should be achieved by increasing either the beam current or the enrichment level.
• **The ‘cool down’ time** - After bombardment, the irradiated water is left to ‘cool’ to allow the decay of shorter-lived radionuclides, such as ^13^N, produced through alternative (*p,n*), (*p,d*) and (*p,* α) reaction pathways as well as due to the resultant intense neutron field from the ^18^O(*p,n*)^18^F reaction [33].
  **Output effect on final tracer properties**
    ▪ **Tracer activity:** longer cooling time will decrease the activity density due to radioactive decay, thus providing lower final water activity density and limiting the maximum activity of the tracers.
    ▪ **Radiochemical purity:** longer cooling time allows the decay of shorter-lived radionuclides produced through alternative and secondary reaction pathways [33], ultimately allowing more accurate initial estimations of the conditions required to achieve a given tracer activity from Eq. (10), and increasing the radiochemical purity of the final tracer, improving its performance.
  **Suggested ranges:** 20–30 min, as the impurities are mainly shorter-lived radionuclides and excessive cooling time will affect the obtainable tracer activity.
    ▪ These impurities can also include longer-lived radiometals sputtered from target materials, though they are present in exceedingly low amounts with activities of the order of kBq [34,35], whereas the resultant total activity is typically of the order of GBq.

For example, the present research team typically employs the following input parameters: a 11.2 MeV beam energy with a 10 *μ*A beam current, and an irradiation time of approximately one hour. For the given level of enrichment of 6.25%, the final activity is between 1 – 2 GBq after an initial ‘cool down’ time of 20–30 min.

The method to obtain the radioactive water is as follows;

Water enriched to the desired quantity with the ^18^O isotope is used as a target.
The water is bombarded with a proton ion beam of specified current and energy for the desired irradiation time, where ^18^O is converted to ^18^F through the ^18^O(*p,n*) ^18^F^−^ nuclear pathway [32].
After bombardment, the irradiated water is left to ‘cool’ for the chosen time.
After the ‘cool down’, all the activity is assumed to be due to the beta decay of ^18^F to ^18^O, and is ready for use in radiolabelling procedures.

The key outputs associated with the radioactive water production method are:

• **Radioactive water with the following key properties:**
  **Total volume** – determines the activity density and number of possible radiolabelling pots/conditions which can be tested in subsequent procedures.
  **Total activity** – measured after bombardment and cool down period
    ▪ determines the activity density for a given volume.
  **Initial activity density** - measured after bombardment and cool down period by dividing the measured activity by the total volume.
    ▪ Can be diluted with distilled water if a large volume of radioactive water is desired and lower individual tracer activities are not an issue.

Low-Temperature Adsorption Radiolabelling Procedure

**Fig. 5:**
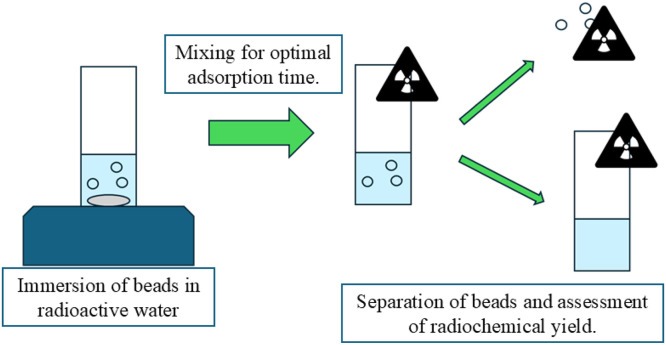
Overview of key steps in low-temperature radiolabelling method.

The cold adsorption method involves the adsorption of ^18^F^-^ ions from radioactive water (produced in accordance with the specifications above) under ambient conditions, where the general procedure is visualised in Fig. 5.

The key input parameters are:

• **Hydrogel beads of given formulation and preparation procedure**
  **Output effect on final tracer properties**
    ▪ **Physical properties:** the final tracer will have the same physical properties as the beads synthesised by the methods specified above.
    ▪ **Radiolabelling properties:** the radiolabelling efficacy and stability will mainly be determined by the bead formulation (chosen gelling cation for solution 2, and chosen MGR, and alginate concentration in solution 1).
• **The initial activity density of radioactive water** – determined by the cyclotron performance and irradiation conditions as specified above.
  **Output effect on final tracer properties**
  **Bead activity:** increases the final bead acitivty for the same volume due to both the large available activity and the increase in concentration of adsorbate in solution (Eq. (8)), which increases adsorption and thus RCY (Henry’s law).
• **Initial water activity** – determined by the activity density of the irradiated water - which depends on cyclotron performance and irradiation conditions as specified above – and the added volume of radioactive water in a single radiolabelling pot.
  **Output effect on final tracer properties**
    ▪ **Bead activity:** increasing the initial activity added will directly increase the final tracer activity.
  **Suggested ranges:** determined from Eq. (10), and bounded by the water activity density and maximum water target volume (Eq. (7)).
• **The volume of radioactive water added**
  **Output effect on final tracer properties**
    ▪ **Bead activity:** for the same initial activity density, increasing the initial volume added will directly increase the final tracer activity.
  **Suggested ranges:** 0.5 – 3 ml
    ▪ Excessive volumes can diminish adsorption yield due to mass transfer limitations, whereas excessively small volumes can become impractical for mixing.
• **Radiolabelling time**
  **Output effect on final tracer properties**
    ▪ **Bead activity:** The radiolabelling time provides a window within which mass transfer of radionuclide from the radioactive water to the bead can occur, increasing the bead activity, whilst potentially decreasing the final bead activity by providing more time for radioactive decay.
  **Suggested ranges:** the time must be sufficient to allow diffusion of radionuclides into the internal pore structure, where excessively short labelling times lead to incomplete adsorption and reduced RCY, while excessively long times deteriorate the achievable activity after the optimal adsorption time (see Fig. 10 below) has been surpassed.
    ▪ The optimal time must be determined for each bead formulation by considering the adsorption kinetics (see the characterisation methods below), but typically the time required is 30 min to 1 h.
• **Number of beads** - The number of tracer particles present in each radiolabelling pot/vessel.
  **Output effect on final tracer properties**
    ▪ **Bead activity:** significantly alters the activity per bead, since the available activity is distributed across all particles.
  **Suggested ranges:** Extremely small values of *n* (e.g., *n* < 3) may reduce the effective RCY due to mass-transfer limitations.
• **Radiochemical yield (RCY)** - depends on both the hydrogel bead formulation and is maximised when radiolabelling experimental conditions are within the optimal ranges as specified above.
  **Output effect on final tracer properties**:
    ▪ **Bead activity**: A lower-than-expected RCY leads to reduced tracer activity and therefore a sub-optimal activity level for PEPT experiments. This decreases the coincidence count rate, which in turn may increase the localisation error during PEPT trajectory reconstruction.

The selection of these input parameters can be guided by considering the estimated final tracer bead activity, $A_{b}$, as calculated from Eq. (10) above. A spreadsheet has also been provided in the supplementary materials, where the above inputs can be altered to observe the change in the final bead activity.

The key steps outlined in Fig. 5 to radio-label hydrogel tracers using the low-temperature adsorption method are as follows:

To prepare the tracers for labelling, the desired number of hydrogel beads are weighed and then placed in a small 5 ml borosilicate glass beaker, along with a micro stir bar of 2 mm in diameter and 7 mm in length.
The desired volume of radioactive water is transferred to the beaker containing the beads using a variable volume micropipette.
The activity at the start of radiolabelling is measured by placing the beaker in a radioisotope calibrator of 0.01 MBq resolution (e.g. a CRC-15 R, Capintec Inc., NJ was used by the present research team [18]), and noting the activity as well as time of measurement.
The beaker is placed on a magnetic stirrer and left under gentle agitation to allow adsorption of the radioisotope for the desired adsorption time.
The total activity in the beaker at the end of adsorption is then measured, taking note of the time.
Labelled tracers are separated from the solution using tweezers and placed in an Eppendorf microtube, allowing the activity of the beads as well as that remaining in the solution to be determined separately.

The key outputs associated with the low-temperature method are:

• **Hydrogel beads radiolabelled with the desired quantity of radioactivity**. These beads can then be employed for:
  Characterization of the radiolabelling properties (RCS and RCY) for novel formulations and/or untested conditions, as discussed below.
  Use in validation studies, as discussed below.
  Use as PEPT tracers for established beads of validated performance.

Evaporative Adsorption Radiolabelling Procedure

**Fig. 6:**
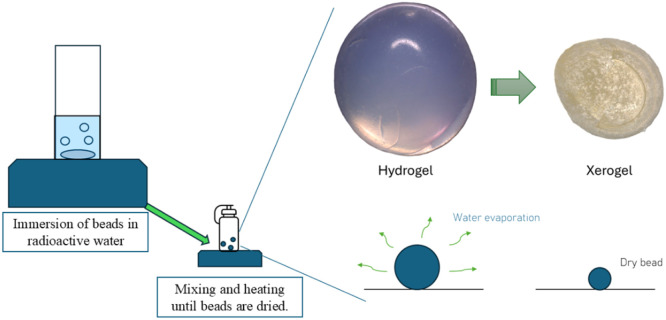
Overview of key steps in evaporative radiolabelling method.

The evaporative adsorption method involves the adsorption of ^18^F^-^ ions from radioactive water (produced in accordance with the specifications above) under heated conditions, where moisture is allowed to escape from the system due to the elevated temperature, resulting in the production of a radiolabelled xerogel (Fig. 6).

The key input parameters are:

• **Hydrogel beads of given formulation and preparation procedure**
  **Output effect on final tracer properties -** the final tracer will have different physical properties to the hydrogel beads, though they will still be dependent on its specific formulation (chosen gelling cation, MGR, and alginate concentration in solution 1) along with the radiolabelling efficacy and stability. In particular, the evaporative method and moisture removal from the xerogel will cause major changes to:
    ▪ **Tracer size:** The initial hydrogel diameter is typically reduced by approximately 60% after evaporative labelling.
    ▪ **True Density:** the final xerogel tracer true density depends on the initial hydrogel bead formulation, but typically increases by 30–80%.
    ▪ **Mechanical properties:** the mechanical stiffness and yield strength of the xerogels is several orders of magnitude greater than that of the hydrogel beads.
    ▪ **Achievable tracer activity:** the RCY achieved under optimal evaporative labelling conditions is typically much greater than in low-temperature methods, regardless of formulation, hence the tracer activity range is also greater and can handle less initial activity density from the cyclotron.
    ▪ **Tracking quality:** because the RCS achieved after evaporative labelling is typically much greater than low-temperature methods, regardless of formulation, the tracking performance of the tracer is generally improved.
• **The initial activity density of radioactive water** – determined by the cyclotron performance and irradiation conditions as specified above.
  **Output effect on final tracer properties**
    ▪ **Bead activity:** will increase the final bead acitivty for the same volume.
• **Initial water activity** – determined by the activity density of the irradiated water - which depends on cyclotron performance and irradiation conditions as specified above – and the added volume of radioactive water in a single radiolabelling pot.
  **Output effect on final tracer properties**
  **Bead activity:** increasing the initial activity added will directly increase the final tracer activity.
  **Suggested ranges:** determined from Eq. (10), and bounded by the water activity density and maximum water volume (Eq. (7)).
• **The volume of radioactive water added**
  **Output effect on final tracer properties**
  **Bead activity:** for the same initial activity density, increasing the initial volume added will directly increase the final tracer activity.
  **Suggested ranges:** 0.5 – 1.5 ml
    ▪ Increasing $V_{w0}$ increases the time required to fully evaporate the water, potentially extending the radiolabelling time and exacerbating decay losses. This can be somewhat alleviated by increasing the temperature, though care must be taken to ensure the mechanical stability of the tracer is not affected due to temperature-induced degradation.
• **Radiolabelling temperature**
  **Output effect on final tracer properties**
    ▪ **Bead activity:** Increasing the temperature decreases the evaporation/radiolabelling time, thus increasing the bead final activity due to less radioactive decay.
    ▪ **Mechanical stability:** Can cause bead embrittlement and degradation.
  **Suggested ranges:** > 40 °C for reasonable evaporation/radiolabelling time, but <100 °C to avoid significant bead degradation/overheating – though embrittlement can typically begin above 70 °C, increasing the likelihood of tracer rupture or attrition in high shear systems.
• **Radiolabelling time**
  **Output effect on final tracer properties**
    ▪ **Bead activity:** decreases the final bead activity through radioactive decay.
    ▪ **Bead physical properties:** increasing the time allows all moisture to be removed and thus convert the hydrogel to a xerogel with different physical properties (see above).
  **Suggested ranges:** For the evaporative method, the time is mainly dictated by the added volume and temperature, as discussed above, and must be sufficient to remove all moisture from the hydrogel bead to obtain the shrunken xerogel tracer.
    ▪ Typically requires 30 – 90 min.
• **Number of beads** - The number of tracer particles present in each radiolabelling pot/vessel.
  **Output effect on final tracer properties**
    ▪ **Bead activity:** significantly alters the activity per bead, since the available activity is distributed across all particles.
  **Suggested ranges:** Extremely small values of *n* (e.g., *n* < 3) may reduce the effective RCY due to mass-transfer limitations.
    ▪ For example, when a single bead is labelled in a flat-bottomed vessel during evaporative adsorption, sub-optimal mixing and restricted contact between the bead and the radioactive solution (e.g. if the bead deposits outside the receding water meniscus during evaporation) may reduce adsorption efficiency.
• **Radiochemical yield (RCY)** - depends on both the hydrogel bead formulation and is maximised when radiolabelling experimental conditions are within the optimal ranges as specified above.
  **Output effect on final tracer properties**:
    ▪ **Bead activity**: A lower-than-expected RCY leads to reduced tracer activity.

The selection of these input parameters can be guided by considering the estimated final tracer bead activity, $A_{b}$, as calculated from Eq. (10) above. A spreadsheet has also been provided in the supplementary materials, where the above inputs can be altered to observe the expected change in the final bead activity.

The general procedure is visualised in Fig. 6, and involves several key steps:

1. To prepare the tracers for labelling, the desired number of hydrogel beads are weighed and placed in a small 5 ml borosilicate glass beaker, along with a micro stir bar of 2 mm in diameter and 7 mm in length.
2. The desired volume of radioactive water is transferred to the beaker containing the beads using a variable volume micropipette.
3. The activity at the start of radiolabelling is measured by placing the beaker in a radioisotope calibrator of 0.01 MBq resolution (e.g. a CRC-15 R, Capintec Inc., NJ was used by the present research team) and noting the activity as well as time of measurement.
4. The beaker is placed on a heated magnetic stirrer at the desired evaporation temperature and left to allow complete evaporation of moisture.
5. The total activity in the pot at the end of adsorption is then measured, taking note of the time.
6. Labelled tracers are separated from the beaker using tweezers and placed in an eppendorf microtube, allowing the activity of the beads as well as that remaining on the walls of the beaker to be determined separately.

**Fig. 7:**
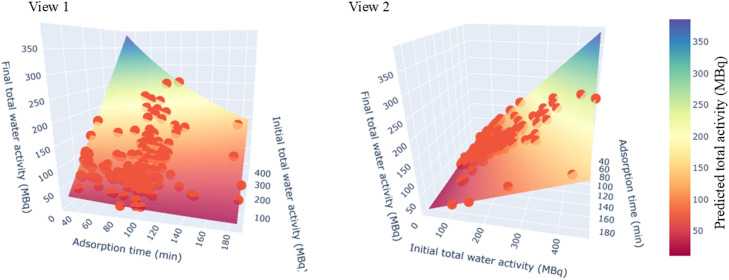
Surface plot demonstrating the agreement between measured and predicted final total activity in the radiolabelling vessel.

**Fig. 8:**
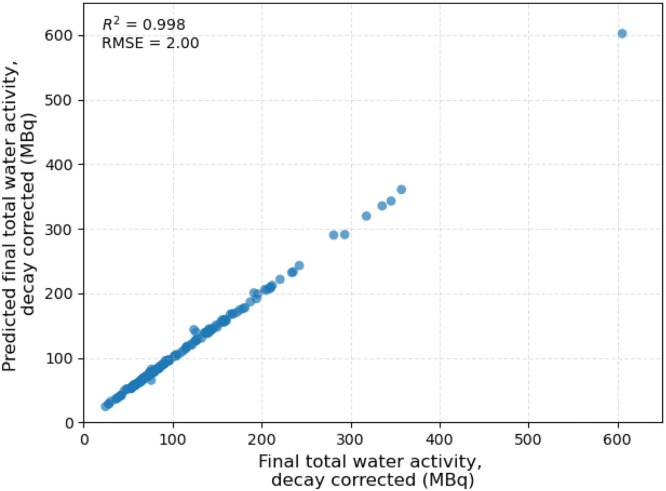
2D plot comparing predicted and measured activity in the pot at the end of the radiolabelling procedure, decay-corrected to a 60-min radiolabelling time.

To ensure the expected activity is achieved at the end of radiolabelling, the whole-pot activity is measured before and after the evaporative radiolabelling procedure (steps 3 and 5, respectively). As visualised in Fig. 7, Fig. 8, this ensures that the activity at the end of radiolabelling matches that predicted by Eq. (9), confirming that no activity (or fluoride ions) are lost to the environment during radiolabelling through, for example, evaporation of moisture.

The key outputs associated with the evaporative method are:

• **Xerogel beads radiolabelled with the desired quantity of radioactivity**. These beads can then be employed for:
  Characterization of the radiolabelling properties (RCS and RCY) for novel formulations and/or untested conditions, as discussed below.
  Use in validation studies, as discussed below.
  Use as PEPT tracers for established beads of validated performance.

Characterisation Methods – Bead Physical Properties

The following characterisation procedures form an essential part of this method as their outputs will advise the selection of certain tracer formulations for different imaging applications and environments. Key parameters to assess for single-particle tracers destined for nuclear imaging include:

• Dry and wet particle mass – to allow further calculation of envelope density and thus porosity (dry particles).
• Dry and wet particle size and morphology – to allow suitable particle sizes/shapes to be selected for different imaging applications, and to allow further calculation of envelope density and thus porosity.
• Dry and wet particle true density – to allow suitable particle densities to be selected for different imaging applications, and to allow further calculation of porosity.
• Dry and wet particle mechanical properties – to ensure suitable particles can be selected for environments involving higher shear.
• Swelling index of dry particles – to ensure the measured dry particle size remains consistent during imaging in fluid media.

Drying of Particles for Characterisation of Xerogels

As a consequence of the evaporative radiolabelling method detailed above, which transforms the hydrogel into a xerogel^I^ through the removal of water, it is important to characterise both the wet (hydrogel) and dry (xerogel) beads in terms of size, morphology, density, and mechanical properties.

To mimic the dehydration conditions in the evaporative radiolabelling process, and thus facilitate the characterisation of larger quantities of dried particles, non-radiolabelled hydrogel beads are dried to obtain xerogels according to the following procedure:

1. Hydrogels are transferred to a glass beaker in a quantity sufficient to form a single layer of beads on the bottom of the glass.
2. The beaker is placed on a hot-plate at the relevant evaporative radiolabelling temperature until incipient dryness is reached.
3. The xerogels are removed from the beaker and stored in airtight containers until further use.

Particle mass and Swelling Index

The mass per bead is determined for both hydrogel and xerogel beads using a scale of 0.1 mg resolution. The procedure involves:

1. Placing enough beads – i.e. enough such that the total mass is at least 100 times the scale resolution – on a pre-tared weighing boat and recording the total mass.
2. Calculating the average mass per bead by dividing the total mass by the number of beads on the scale.

Measurements are performed in triplicate for both hydrogels and xerogels to determine the standard deviation and quantify weight loss upon drying.

The swelling index (SI) of the dried xerogels can then be obtained. For this assessment, the gravimetric method is employed [36,37] as follows:

1. The dry mass of enough xerogel particles – such that the total mass is at least 100 times the scale resolution – is weighed on a pre-tared weighing boat.
2. The weighed beads are submerged in the swelling medium of interest within a sealed container.
3. The beads are left to swell at ambient temperature for the desired assessment time.
• For example, a swelling time of 48 h is specified by the Japanese Industrial Standard K8150 [38] and can serve as a reference for comparison with other studies reporting alginate bead swelling.
• Given that the half-life of ^18^F is 109.7 min, and that the allowable single-particle activity for PEPT is rarely > 100 MBq (depending on the detector system), whilst PET imaging procedures are typically conducted within one hour [39], it may be more meaningful to evaluate the SI over timescales on the order of hours rather than days in some instances.
4. After the desired swelling time, the beads are isolated individually using precision tweezers and placed on paper towel to remove loosely bound surface moisture. The swelled beads are then weighed again, allowing the SI to be obtained as follows:
  (11) $$Swelling\;Index,\;SI\left(\%\right)=\frac{m_{s}-m_{d}}{m_{d}}\times 100$$

where $m_{s}$ is the mass of the swelled beads, and $m_{d}$ is the mass of the initial dry beads [36,38].

Size and Shape Analysis

**Fig. 9:**
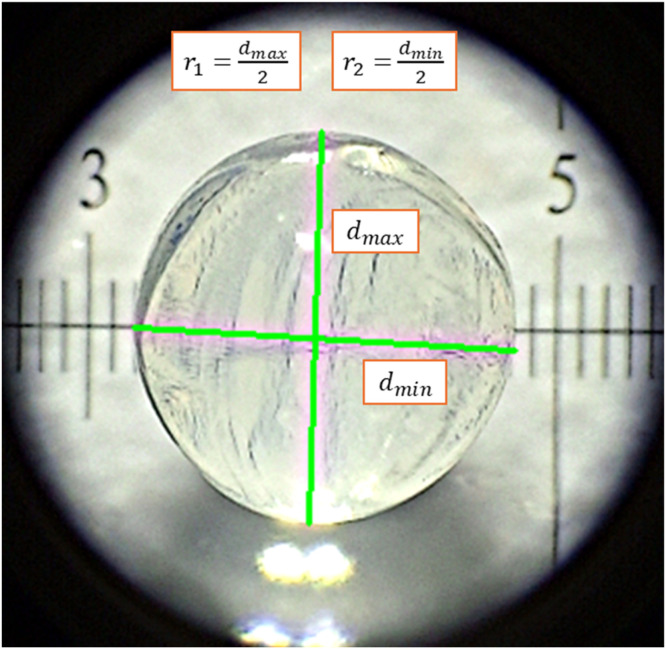
Example of image analysis procedure for hydrogel beads (from the top view — see text for details).

The size of spherical hydrogel beads can be characterised using any suitable optical light microscope (e.g. a Leica DM 2500 LED was used in the related research article [18]). At least enough beads to ensure a standard error of < 5% are captured by placing the hydrogel particles under the microscope either individually or in groups (so long as the individual beads are not in contact), with the following specific procedure:

1. Before imaging, the surface moisture is removed from the hydrogel beads by briefly drying on a paper towel – note that the hydrogels should not be left out of an aqueous environment for >5 min before imaging to avoid shrinkage due to moisture loss.
2. If the hydrogels are obviously deformed in one plane then each bead should be used to generate two images, one in the "top view" plane, and one in the "side view" plane, allowing accurate estimation of the true maximum and minimum diameters, as demonstrated in Fig. 9.

**Fig. 10:**
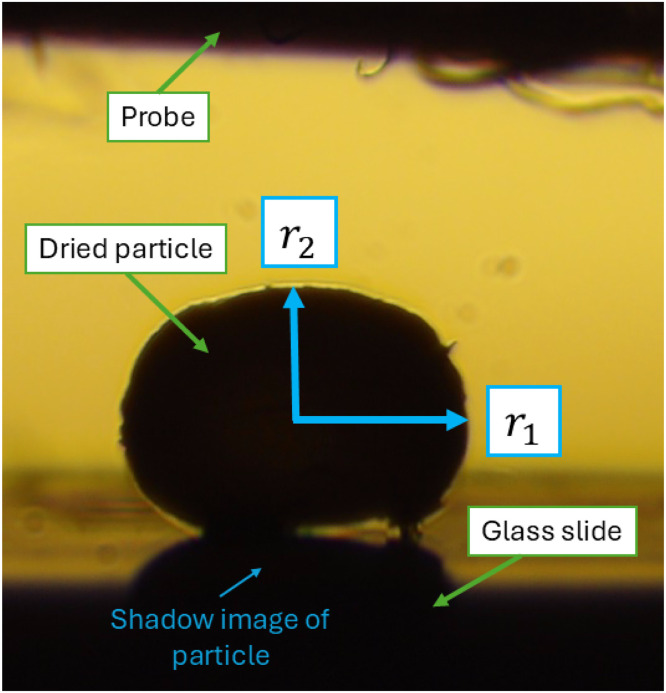
Example of image analysis procedure for xerogel beads.

The xerogel particles obtained after drying are generally less spherical than their swollen hydrogel counterparts and more difficult to manoeuvre under the optical microscope. While the top view typically retains a high degree of sphericity, the side view often appears more eccentric. For this reason, size characterisation of xerogel particles must be carried out from the side view plane. This can be integrated with the mechanical characterisation procedure using the micromanipulation technique described below. The micromanipulation rig is equipped with a side-view camera, enabling measurement of both the equatorial and polar radii, as illustrated in Fig. 10.

The images obtained can be analysed using conventional image analysis techniques, such as imageJ software or image processing in python [19,[40], [41], [42]]. The main metrics to be extracted are the maximum and minimum radii, $r_{1}$ and $r_{2}$, allowing the particle volume, *V*, to be calculated, assuming all particle shapes can be approximated by either an oblate spheroid, a prolate spheroid, or a perfect sphere to account for those which may appear distorted in one dimension or irregular in shape. The approximate shape characteristic of each formulation is evaluated before the analysis, where the relevant shape parameter calculations are specified in Table 3. The calculations in Table 3 enable the equivalent diameter, simplified sphericity index, and eccentricity to be obtained and provide useful metrics for the particle shape and morphology [43].

**Table 3:** Particle shape characterisation calculations.

Shape	Sphere	Prolate spheroid	Oblate spheroid
$r_{1}$	Largest measured radius	Largest radius, or “polar” radius	Largest radius, or “equatorial” radius
$r_{2}$	Smallest measured radius	Smallest radius, or “equatorial” radius	Smallest radius, or “polar” radius
*Volume*	$V=\frac{4}{3}\pi {\left(\frac{r_{1}+r_{2}}{2}\right)}^{3}.$	$V=\frac{4}{3}\pi r_{1}r_{2}^{2}.$	$V=\frac{4}{3}\pi r_{1}^{2}r_{2}.$
*Equivalent diameter*	$D_{e}={\left(\frac{6V}{\pi }\right)}^{1/3}$		
*Sphericity index)*	$\phi =\frac{D_{e}}{2r_{1}}$		
*Eccentricity*	$\sqrt{1-\frac{r_{2}^{2}}{r_{1}^{2}}}$		

### Density measurement

#### True density

The true density of both hydrogels and xerogels can be measured using any helium gas pycnometer [[44], [45], [46]] capable of handling semi-solid samples, with active temperature control, and bi-directional gas expansion capability (an Ultrapyc 5000 micro, Anton Paar was used in the related research article [18]), ideally equipped with a relatively small sample holder (< 2 cm^3^) to facilitate measurements with small sample sizes. Sample preparation involves:

1. Pre-tareing the sample holder on any scale of 0.1 mg resolution.
2. Filling the sample holder with the available quantity of hydrogel or xerogel until at least 50% full by visual inspection.
  For hydrogel beads, surface moisture should be removed by briefly drying with a paper towel.
3. Weighing the filled amount by reading off the scale - the measured sample mass is then used as input to the pycnometer for density calculation.
4. The sample holder is then placed in the test cell such that the density can be characterised at 25 °C.

Since the helium gas cannot penetrate the waterlogged pores of hydrogel particles (as evinced by its common use in the determination of material water content [47]), the density determined through helium pycnometry is taken as representative of the hydrogel envelope density, where envelope density refers to the density of the particle including the volume of its internal pores and voids [48], and is equivalent to the true hydrogel density. However, the dry xerogels do allow helium penetration into the internal structure, hence this characterisation method provides only the true density, defined as the density of the particle skeleton, excluding internal pores and voids [48].

#### Envelope density and porosity (only xerogels)

The envelope density of dry xerogel particles is quantified using the particle size data obtained via image analysis (as specified above). This allows the volume of each dry particle, $V_{d}$, to be calculated, assuming an approximate particle shape from Table 3. The envelope density is thus obtained through division of the dry particle mass (determined through the procedure specified above) by the calculated volume. This also allows the apparent porosity, *φ*, of the xerogels to be estimated as follows:

(12) $$\varphi =1-\frac{\rho_{e}}{\rho_{t}}$$

where $\rho_{e}$ and $\rho_{t}$ are the respective envelope and true density (where $\rho_{t}$ is determined by the procedure specified above) of the xerogel particle [49].

### Mechanical analysis

The mechanical analysis involves compressive testing of the hydrogel and/or xerogel beads using a computer-controlled mechanical testing system capable of measuring force and displacement during controlled deformation of a single bead sample. The correct mechanical testing equipment will depend on both the form (hydrogel or xerogel) and size of the specimen, since both these parameters affect the required instrument sensitivity. Thus, the following describes the methods for hydrogels and xerogels separately.

#### Hydrogels

**Fig. 11:**
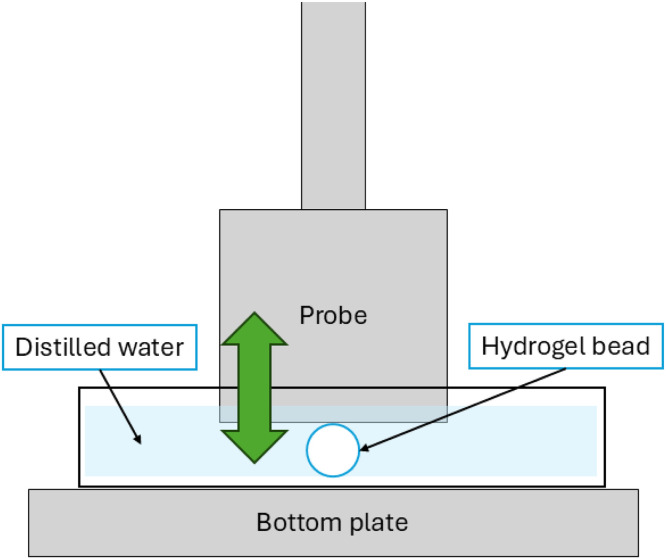
Depiction of the compression testing set-up to determine mechanical properties of wet hydrogel beads.

The mechanical characterisation of large (> 1 mm) hydrogel particles is performed using any universal testing machine with a force resolution of at least 0.1 g, such as a Texture Analyser (a TA.XTplusC Texture Analyser from Stable Micro Systems, equipped with a 5 kg load-cell - force resolution of 0.1 g, sensitivity of 1.0 ± 0.15 mV/V – and a 40 mm cylinder probe attachment was used in the related research article [18]). A flat bottomed cylindrical probe is used to compress single beads to obtain both the Young’s Modulus (E) and strain at break (SB). The general set-up of this measurement is demonstrated in Fig. 11.

The measurement is conducted through the following method:

1. To prepare for the measurement, a petri dish of much larger diameter (> 2 times the probe diameter) than the cylinder probe is centred on the TA platform.
2. The probe height is calibrated to the petri dish surface.
3. The dish is filled with distilled water, such that the fluid level is at least 3-fold higher than the bead diameter.
4. A hydrogel particle is centred beneath the probe in the m50le of the petri dish.
5. The probe is carefully lowered into the fluid and set at a start height hovering a few millimetres above the bead.
6. A compression test is conducted, whereby the probe compresses the bead to a given strain before returning to its start point.
➢ The typical test parameters used in the related research article [18], which align with those employed in similar studies from the literature for the characterisation of alginate-based hydrogel beads [50,51], are given in Table 4, where "low strain" measurements are used to obtain *E*, and "high strain" measurements are used to evaluate the SB.

**Table 4:** Parameters used for hydrogel compression testing.

Parameter	Value	Units	Ref.
*Pre-test probe speed*	40	mm/min	[50,51]
*Test speed*	40	mm/min	[50,51]
*Post-test speed*	40	mm/min	[50,51]
*Target strain*	Low strain: 30 / high strain: 99	%	[50,51]
*Trigger force to begin measurement*	1	*g*	-
➢ Importantly, the compression speed should be relatively high – at least 40 mm/min, as recommended in the literature on hydrogel bead mechanical testing to avoid the influence of viscoelastic effects [50,51].
7. Each measurement should be repeated at least 5 times or ideally > 10 depending on the variability in the observed mechanical response, using a different bead for each test.

The force vs displacement data are typically stored in a spreadsheet, and can be analysed either manually in Excel or via a python script. Here, *E* is calculated at nominal strains below 30%, since Chan et al. (2011) [50] have reported reversible and instantaneous recovery below this limit, where the nominal strain, *ε*, is defined as:

(13) $$\varepsilon =\frac{H}{H_{0}}\times \;100,$$

where $H_{0}$ is the initial height of the probe at the start of the measurement, and *H* is the bead displacement. This allows evaluation of *E* using classical Hertzian theory for spherical geometries;

(14) $$F=\frac{4r^{1/2}}{3}\frac{E}{1-v^{2}}{\left(\frac{H}{2}\right)}^{3/2};$$

where *F* is the force measured by the probe, *r* is the initial bead radius (before compression), and *v* is Poisson’s ratio^II^ [52,53]. Hence, a plot of *F* against ${\left(\frac{H}{2}\right)}^{3/2}$ should yield a straight line, where the slope, *m*, allows *E* to be obtained as follows;

(15) $$E=\frac{3m\left(1-v^{2}\right)}{4r^{1/2}}.$$

**Fig. 12:**
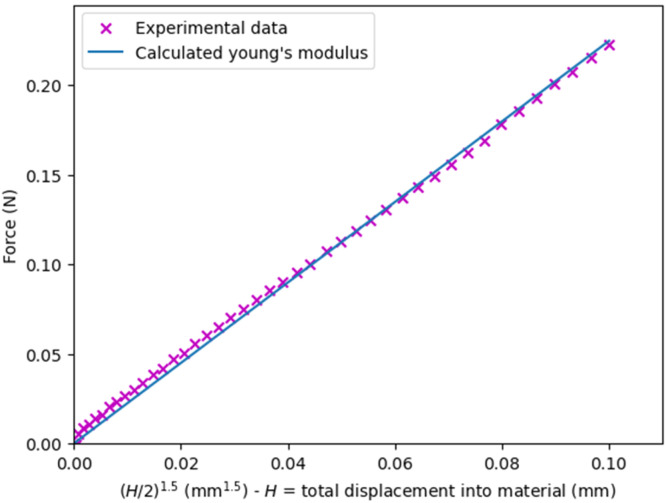
Example typical fitting of Young's modulus below 30% nominal strain of hydrogels – the formulation to which the measurement corresponds is an initial solution of 4 wt.% sodium alginate of high MGR (M/G = 1.56), dripped into a lanthanum chloride solution of 8 wt.% concentration using a 0.21 mm ID nozzle (27 G), with an approximate final equivalent diameter of 2.38 mm. The coefficient of determination (R^2^) in this case was 0.997.

This is demonstrated in Fig. 12.

**Fig. 13:**
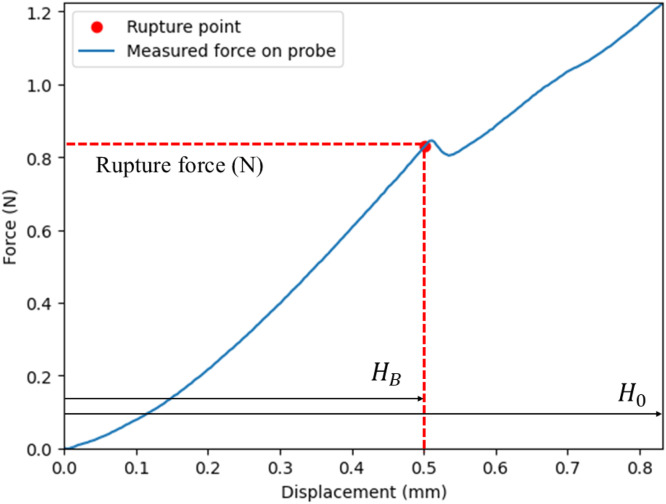
Example evaluation of strain at break (SAB) for hydrogel beads, where $H_{0}$ is the initial height of the probe at the start of compression, $H_{B}$ is the displacement at break, and thus $SAB\;\left(\%\right)=\frac{H_{B}}{H_{0}}\times \;100$.The formulation to which the measurement corresponds is an initial solution of 4 wt.% sodium alginate of low MGR (M/G = 0.59), dripped into a lanthanum chloride solution of 8 wt.% concentration using a 0.21 mm ID nozzle (27 G), with an approximate final equivalent diameter of 2.07 mm.

The SB refers to the fractional deformation required to cause rupture of the hydrogel bead, and is obtained by compressing up to 99% with respect to the initial sample height and observing the strain at which catastrophic failure occurs, as demonstrated in Fig. 13. In some cases - i.e. for elastic, durable, and ductile gels - a rupture point is not observed, and the bead returns to its original shape after compression.

#### Xerogels

As aforementioned, the xerogels are produced through dehydration and thus shrinking of the hydrogel beads at moderately high temperature, resulting in beads with diameters approximately 60% less than that of their hydrogel precursors, and thus often < 1 mm. Due to this difference in hydrogel vs xerogel particle size range, a more specialized instrument of higher sensitivity is required to reduce the noise-to-data ratio [54]. The micromanipulation techniqueIII is thus required to characterise the mechanical properties of the xerogels, though it should be noted that this equipment is unsuited for beads much greater than 1 mm due to concerns over damaging the highly sensitive force transducer [55]. Nevertheless, the underlying principles of force measurement under compression to assess the mechanical properties of the beads remain consistent across both hydrogel and xerogel characterisation methods [56].

The micromanipulation rig used for xerogel characterisation has been described previously [562]. During equipment operation, samples are diametrically compressed between two parallel surfaces and the force response is recorded via a probe connected to a force transducer. The method to obtain this data is as follows:

1. Prior to use, the compliance of the probe is calibrated to account for its displacement under a given compressive force. This correction allows accurate measurement of the force required to achieve a specified particle deformation.
2. A single xerogel bead is manually placed on a flat glass slide in ambient conditions using tweezers, making sure to use minimal force to move the tracer so as not to damage the particle before measurement.
3. The particle is compressed using a flat-end cylindrical probe connected to a force transducer. The compression is continued until the full force load of the transducer is applied to the xerogel.
➢ As a reference, the equipment and transducer specifications used in the related research article [18] are given in Table 5.

**Table 5:** Micromanipulation equipment specifications used in the related research article [18].

Component	Metric	Value
Glass side	Area	2.5 cm^2^
Probe	Diameter	1 mm
Transducer	Manufacturer	Aurora Scientific Inc., Canada
	Model	402,001
	Series	402A
	Maximum load	500 mN
	Sensitivity	45.894 mN/V
4. The raw dataset is recorded to generate the corresponding force vs displacement curves, allowing subsequent application of Eq. (14) for quantification of *E* below the elastic limit,^IV^ as well as evaluation of other relevant properties (e.g. fracture toughness and rupture force, among others [52]).
5. This is repeated for at least 15 different xerogel particles to obtain an accurate assessment of the standard error.

### Characterisation of radiolabelling properties

#### Evaluation of cold adsorption radiolabelling kinetics

The kinetics of the cold adsorption method can be assessed to determine both the rate of fluoride uptake and inform the optimal adsorption time required for the low-temperature radiolabelling process. This is achieved by modifying the low-temperature labelling process methodology as follows:

To prepare the tracers for labelling, the desired number of hydrogel beads are weighed and then placed in a small 5 ml borosilicate glass beaker, along with a micro stir bar of 2 mm in diameter and 7 mm in length.
The desired volume of radioactive water is transferred to the beaker containing the beads using a variable volume micropipette.
The activity at the start of radiolabelling is measured by placing the beaker in a radioisotope calibrator of 0.01 MBq resolution (e.g. a CRC-15 R, Capintec Inc., NJ was used by the present research team), and noting the activity as well as time of measurement.
The beaker is placed on a magnetic stirrer and left under gentle agitation to allow adsorption of the radioisotope for sufficient time to observe a ‘plateau’ in the kinetics data.
At each time interval small aliquots of radioactive solution are withdrawn from the adsorption vessel.
  ➢ The time interval is typically 10 min, but can be varied to achieve more or less continuous kinetic data, where the most suitable value depends on the adsorption rate.
  ➢ The aliquot volume should be chosen on a case-by-case basis, depending on total solution volume and activity concentration - i.e. the volume should be large enough to provide detectable activity and a representative measurement, but should not be so small that it introduces errors due to losses upon transfer from the adsorption vessel to the measuring vessel.
The time of measurement and activity of the aliquot is measured with the radioisotope calibrator.
After each measurement, the aliquot is returned to the radiolabelling vessel, and the procedure is repeated following another time interval.
The total activity in the beaker at the end of adsorption is then measured, taking note of the time.

**Fig. 14:**
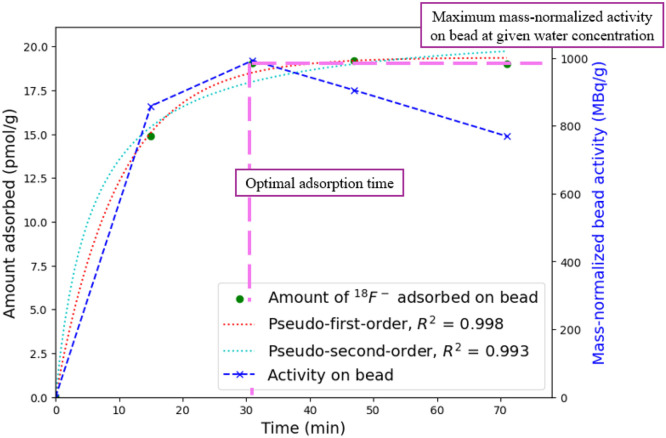
Example evaluation of optimal adsorption time for low temperature adsorption method.

The molar concentration of fluoride remaining in the adsorbate solution, $C_{s}$, at each time interval is thus calculated from Eq. (8). These values are used to evaluate the amount of ^18^F^-^ anions adsorbed on the bead at each time interval, taking into account the decay of ^18^F^-^ to ^18^O. The total fluoride uptake is thus plotted against time, and fit to relevant kinetic models, such as the Pseudo-First-Order (PFO) and Pseudo-Second-Order (PSO) equations. The variation of activity on the beads at each time interval can also be visualised, allowing assessment of the optimal adsorption time for maximum bead activity - i.e. the point at which the rate of ^18^F decay surpasses the rate of activity increase due to the adsorption of active fluoride ions, as demonstrated in Fig. 14.

#### Radiochemical yield

The radiochemical yield (RCY) achieved after either low temperature of evaporative radiolabelling can be determined following the method of Pellico et al. (2022, 2024) [12,57];

(16) $$RCY=\frac{A_{b}}{A_{b}+A_{p}};$$

where $A_{b}$ is the activity of the bead after radiolabelling, and $A_{p}$ is the remaining activity in the pot used for radiolabelling (i.e. the activity remaining in the adsorbate solution and the activity remaining on the wall of the empty beaker and stirrer respectively in the low-temperature and evaporative methods described above). The time delay in measurement (due to the need to remove the beads from the pot and measure each separately) is taken into account using Eq. (9), such that all activities are ‘decay corrected’ to the same final adsorption end-point.

It should be noted that different researchers may have different definitions of RCY. For example, some works in the literature use a ‘decay-corrected’ RCY, which evaluates the yield by considering the ratio of the final activity to the initial activity produced from the cyclotron target [58]. To be clear, the definition provided in Eq. (16) is ‘uncorrected’ for radioactive decay with respect to the initial cyclotron output, solely considering the percentage of fluorine atoms adsorbed onto the radiolabelled material as compared to those remaining in solution. This facilitates comparison to previous works which discuss solid particle radiolabelling for PET and PEPT applications [57].

#### Measurement and evaluation of radiochemical stability

The radiochemical stability (RCS) is determined after either low-temperature or evaporative radiolabelling of a given tracer formulation via the following methodology:

1. Isolated tracers are placed in a 1.5 ml polypropylene microcentrifuge tube.
2. 1 ml of distilled water is pipetted into the tube, ensuring all particles are submerged in the washing fluid.
3. The tube cap is closed and the vial is vortexed for 30 s using a vortex mixer.
4. The fluid is pipetted out of the vial, making sure not to entrain any of the particles in the pipette nozzle.
5. The above process is repeated thrice to ensure all loosely adsorbed surface activity is removed.
6. After rinsing, the remaining activity is again measured to quantify the extent of loosely bound surface adsorption.
7. The tracers are placed in a small glass vial of distilled water and left to stir for 1 to 3 h at room temperature, depending on the initial activity. Tracers can be left for a longer amount of time if the remaining activity is high enough to ensure an accurate reading at the end of the stability test.

The RCS is measured following the method of Pellico et al. (2022) [57], which is quantified as follows:

(23) $$RCS=\frac{A_{b}}{A_{b}+A_{w}};$$

where in this case $A_{b}$ is the activity of the bead after RCS testing, and $A_{w}$ is the activity of the remaining water.

## Method validation

### Validation framework

The performance of novel alginate tracers - fabricated, characterised, and radiolabelled via the methods described above - as PEPT tracers can be validated by comparing the tracking behaviour and ability to reconstruct flow characteristics with that of a previously established tracer with known efficacy in PEPT measurements. Conventional ion-exchange resin (IER) tracers are currently among the most widely employed surrogate particles in PEPT studies and therefore provide a suitable control for this purpose.

**Fig. 15:**
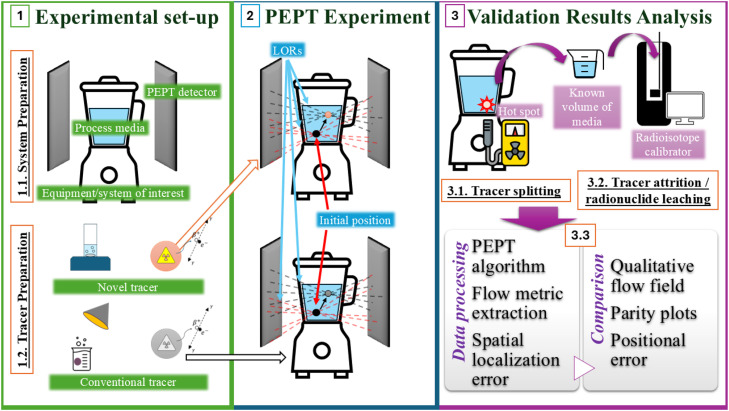
Validation of tracers via comparative PEPT experiments.

**Fig. 16:**
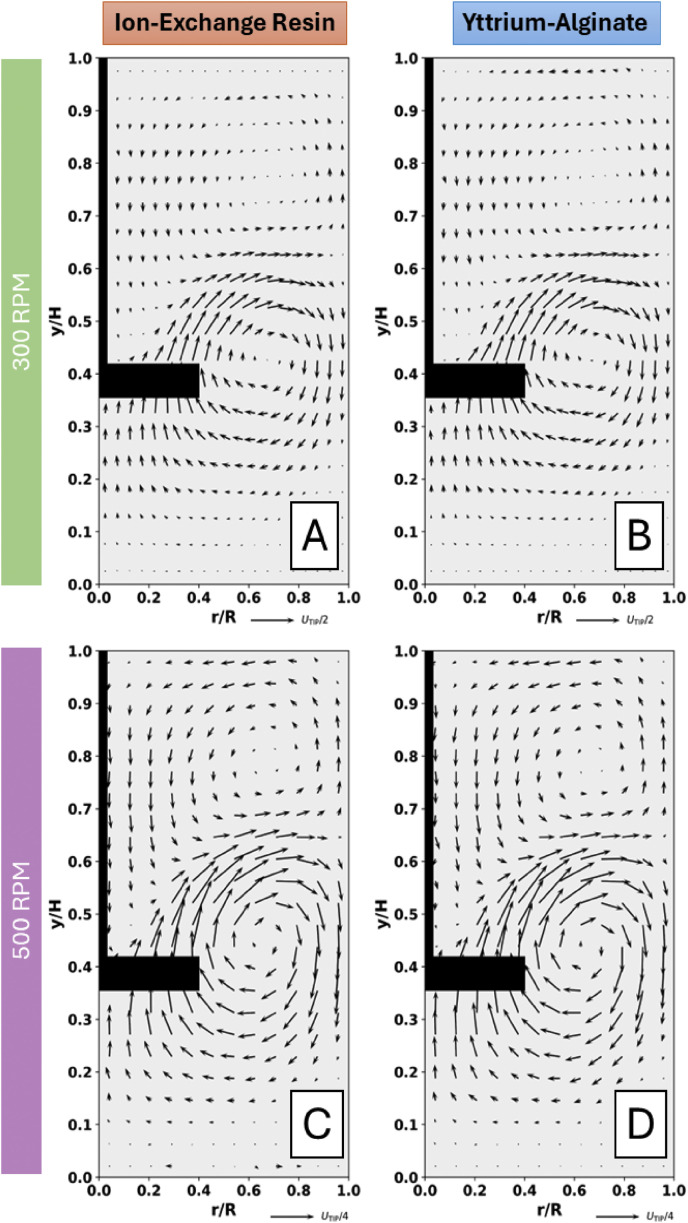
Azimuthally averaged velocity vector fields obtained inside the stirred tank setup at 300 RPM from PEPT data using A) an ion exchange resin tracer, and B) novel yttrium-alginate tracers presented in this work, as well as at 500 RPM from PEPT data using C) an ion exchange resin tracer, and D) novel yttrium-alginate tracers presented in this work. The specifications of the mixing tank system used are as follows: a cylindrical flat-bottomed tank of 0.2 m in diameter, with a fluid height to diameter ratio of 1, corresponding to a total fluid volume of 6.283 L. The vessel contains four rectangular baffles equally spaced along the wall perimeter, each with a width of 2 cm. An IKA Eurostar 200 Control overhead stirrer was employed to rotate the impeller shaft, which was equipped with a 60° up-pumping pitched blade turbine (PBT), of 8 cm diameter positioned at a clearance height of 7.2 cm.

**Fig. 17:**
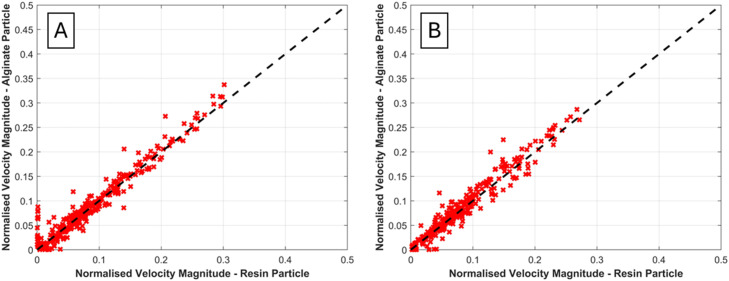
Parity plots of the normalised velocity for both yttrium-alginate and ion-exchange resins for an impeller speed of A) 300 RPM, and B) 500 RPM.

**Fig. 18:**
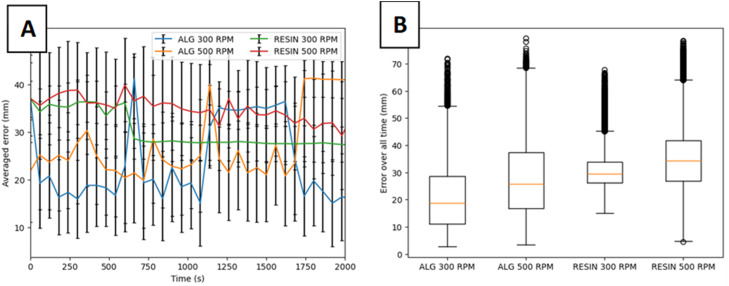
A) Error in positional accuracy over time, with error averaging over a timestep of 1 min, B) boxplot to compare the error over the whole experimental window.

In such a validation study, PEPT experiments are performed under identical operating conditions using both the novel alginate tracer and a conventional control tracer. The resulting datasets can then be compared to determine whether the novel tracer reproduces equivalent particle trajectories and derived flow metrics within the system of interest. These comparisons may include, for example, velocity fields, particle occupancy distributions, shear rate fields, or other statistical quantities. The general workflow of this validation study is illustrated schematically in Fig. 15, which outlines both the experimental preparation and the subsequent data evaluation stages, the methodology of which is elaborated in the subsequent text. An example of such a validation procedure, together with the associated experimental configuration, is described in greater detail in the related research article [18] for a stirred tank system, and is presented here in Figs. 16- 18 to serve only as an illustrative example, demonstrating the workflow and evaluation methods application. The validation framework developed here is system-agnostic and can be applied to any PEPT-imaged environment.

## Validation study experimental set-up

The validation study begins with preparation of the experimental system for PEPT measurements (Fig. 15).

### Preparation of the system

1. Determine the most appropriate detector system for the component or equipment to be imaged.
  Guidelines to assist this selection can be found in [6] and [8].
  The choice of detector influences several experimental parameters, including:
    ▪ **Optimal tracer activity,** due to the variation in maximum coincidence acquisition rates of different detector systems.
    ▪ **Field of view**, which is particularly important when imaging large vessels or extended flow domains.
    ▪ **Imaging quality/accuracy**, including the proportion of false coincidence events that may be recorded.
  In general, detector systems with higher coincidence acquisition rates enable improved tracking of rapidly moving particles but require higher tracer activities. Conversely, larger detector arrays provide an increased field of view but may exhibit reduced spatial resolution.
2. Position the equipment within the detector field of view such that all regions of interest are captured during the measurement.
3. Fill the system with the required volume of non-radioactive process media so that the experiment can be initiated immediately after introduction of the tracer particle.

Once the experimental system is prepared, the tracer particles can be activated.

### Preparation of the tracers

*Alginate tracer (validation particle)*

1. Select the tracer formulation and radiolabelling method to be validated for the system.
2. Determine the required radiolabelling parameters to achieve the optimal tracer activity for the chosen detector system from the guidelines below.
3. Radiolabel the tracer via either the low-temperature method or the evaporative method described in the preceding sections.

*Control tracer*

A conventional tracer should be prepared and radiolabelled in a duplicate experiment to provide a baseline for comparison.

• For example, established methods for the radiolabelling of ion-exchange resin particles for PEPT measurements are described by Leadbeater et al. (2023) [13].

#### Activity prediction and sensitivity analysis

***Activity Prediction***

If the approximate radiochemical yield (RCY) for a given tracer formulation and radiolabelling method is known, the final tracer activity can be estimated using:

$$A_{b}=\text{RCY}\;\times \frac{A_{w0}\text{exp}\left(-\lambda t\right)}{n},$$

where $A_{b}$ is the predicted activity of an individual tracer bead after radiolabelling, RCY is the radiochemical yield of the formulation for a given radiolabelling process, $A_{w0}$ is the initial activity of the radioactive water introduced to the radiolabelling vessel, n is the number of beads undergoing radiolabelling in the same vessel, t is the radiolabelling time, and λis the radionuclide decay constant. The initial water activity can be calculated from the activity density and added volume:

$$A_{w0}=\rho_{A,w0}V_{w0}$$

where $\rho_{A,w0}$ is the volumetric activity density of the irradiated water, and $V_{w0}$ is the volume of radioactive water used to radiolabel the tracers. Comparison of the predicted bead activity with the experimentally measured activity provides a useful validation of the radiolabelling process and can identify potential inefficiencies arising from incomplete adsorption, tracer handling losses, or deviations from the expected radiochemical yield.

***Sensitivity Analysis of Radiolabelling Parameters***

The above also enables sensitivity analysis of the radiolabelling process by examining how the predicted bead activity varies with key experimental parameters. The most important parameters influencing tracer activity are:

• **Initial water activity,** $A_{w0}$ - This parameter has the strongest influence on final bead activity and can be varied by adjusting the volume of radioactive water added during radiolabelling. The achievable value is constrained by:
  the activity density of the irradiated water ($\rho_{A,w0})$, which depends on cyclotron performance and irradiation conditions.
    ▪ For example, the activity density of the radioactive water produced using the MC40 cyclotron at the University of Birmingham usually varies between approximately 0.3 −1 GBq/ml, though this will depend highly on the type of cyclotron and other important parameters, as described in the methods.
  the volume of water added ($V_{w0}$), which must remain compatible with the chosen radiolabelling method.
    ▪ For the evaporative adsorption method, increasing $V_{w0}$ increases the time required to fully evaporate the water, potentially extending the radiolabelling time and increasing decay losses. This can be somewhat alleviated by increasing the temperature, though care must be taken to ensure the mechanical stability of the tracer is not affected due to temperature-induced degradation.
    ▪ For the low-temperature adsorption method, excessive volumes can diminish adsorption due to mass transfer limitations.
• **Radiolabelling time,** t - The radiolabelling time influences the final bead activity through radioactive decay. Short radiolabelling times are desirable to minimise decay losses; however, sufficient time must be allowed for adsorption of the radionuclide onto or into the tracer particle.
  For hydrogel-based tracers, the time must be sufficient to allow diffusion of radionuclides into the internal pore structure. Excessively short labelling times (depending on the adsorption kinetics of the given formulation) may therefore lead to incomplete adsorption and reduced RCY, while excessively long times deteriorate the achievable activity after the optimal adsorption time (Fig. 14) has been surpassed.
  For the evaporative method, the time is mainly dictated by the added volume, as discussed above.
• **Number of beads,** *n* - The number of tracer particles present during radiolabelling provides a simple method for adjusting the activity per bead, since the available activity is distributed across all particles. However, extremely small values of *n* (e.g., *n* < 3) may reduce the effective RCY due to mass-transfer limitations.
  For example, when a single bead is labelled in a flat-bottomed vessel during evaporative adsorption, sub-optimal mixing and restricted contact between the bead and the radioactive solution (e.g. if the bead deposits outside the receding water meniscus during evaporation) may reduce adsorption efficiency.
• **Radiochemical yield (RCY)** - The RCY represents the dominant source of uncertainty in the activity prediction and depends on both the tracer formulation and the radiolabelling method employed (e.g., low-temperature or evaporative adsorption). As is evident from the above, the RCY may also be influenced if sub-optimal experimental conditions are chosen such as:
  Extremely low or high values of $V_{w0}$.
  Extremely low values of n.
  Excessively short radiolabelling time.

A lower-than-expected RCY leads to reduced tracer activity and therefore a sub-optimal activity level for PEPT experiments. This decreases the coincidence count rate, which in turn may increase the localisation error during PEPT trajectory reconstruction.

***Practical Implementation***

In practice, the predicted tracer activity calculated using the above equations can be compared with the activity measured using a radioisotope calibrator following radiolabelling. Agreement between predicted and measured values provides additional validation of the radiolabelling process and allows identification of systematic deviations in RCY for a given tracer formulation.

To facilitate implementation of the activity prediction calculation, the above equations have been simplified in the spreadsheet provided in the supplementary materials.

## PEPT data acquisition

Following radiolabelling, the tracer is introduced into the system and allowed to circulate within the flow field. PEPT data are simultaneously acquired for a sufficient duration to obtain statistically representative particle trajectories and ensure ergodic sampling of the system. Important considerations during this stage include:

• Ensuring that both the control tracer and the tracer under validation are introduced into the system in a consistent manner to minimise variability arising from differing initial conditions.
• The optimal acquisition time:
  PEPT experiments typically run for 1 - 3 h, depending on factors such as the coincidence acquisition rate of the detector system, tracer activity, and the dynamics of the flow being investigated. Systems exhibiting rapid internal dynamics or strong attenuation may also require longer acquisition times.
  If desired, the optimal acquisition time can be estimated using pre-experimental GATE simulations, as described by Herald et al. (2021) [59].

## Validation study analysis

The results obtained from the validation experiments can then be evaluated to verify the efficacy of the synthesised and radiolabelled tracer. Before undertaking detailed comparisons of PEPT-derived flow metrics, several preliminary checks can be performed to confirm tracer stability within the system.

### Tracer mechanical robustness

The tracer should remain intact within the shear environment of the system of interest. Mechanical failure may occur through catastrophic fragmentation of the tracer particle into two or more individual pieces. The presence of fragmentation, or “tracer splitting”, can be assessed by scanning the equipment using a Geiger counter:

• If the tracer remains intact, a single localized region of radioactivity (a single “hot spot”), where the Geiger counter exhibits continuous auditory output, should be detected.
• If fragmentation has occurred, multiple hot spots corresponding to individual tracer fragments will be observed.

### Tracer mechanical and radiochemical stability

The tracer should not leach significant radioactivity into the surrounding media through desorption or mechanical attrition. The extent of activity release can be estimated by:

• Collecting a known quantity (volume or mass basis) of the process media after the PEPT experiment (excluding the tracer particle).
• Measuring the activity of this sample using a radioisotope calibrator to determine the activity density.

This activity density can be used to estimate the total activity released into the system, given that the total volume/mass of media introduced at the start of the experiment is known. The total activity detected in the non-radiolabelled media should be consistent with the expected radiochemical stability (RCS) of the tracer formulation.

• For example, if the tracer RCS was previously determined to be 90% from the above methods, and the tracer activity after the PEPT experiment is 9 MBq, then the activity present in the surrounding media should be less than approximately 1 MBq.

It should be noted that this procedure does not directly distinguish between desorption and mechanical attrition mechanisms – i.e. it will be unclear if there is leaching due to mechanical wear on the tracer or the cleavage of chemical/physical bonds between the tracer material and the radionuclide. However, insight may be obtained from the mechanical characterisation of the tracer material described in the methods section. For example, more brittle tracer formulations with high Young’s modulus may be more susceptible to activity loss through particle attrition, whereas more elastic particles are less likely to undergo mechanical wear.

### Validation of PEPT data using the novel tracer

If the tracer is shown to remain stable within the system of interest, further analysis of the PEPT dataset can be undertaken to compare its performance with that of the conventional control tracer. Raw PEPT data consist of the x-y coordinates corresponding to the start and end points of each detected line-of-response (LOR), together with the timestamp of the coincidence event. These two-dimensional data can be processed using a suitable PEPT reconstruction algorithm to determine the three-dimensional particle trajectory as a function of time, where guidelines for the selection of appropriate PEPT algorithms and trajectory reconstruction methods are provided by Windows-Yule et al. (2022) [6,8]. The reconstructed trajectories can subsequently be analysed to derive a range of flow metrics, such as particle velocity fields, spatial occupancy distributions, residence-time statistics, or shear rate fields. Comparisons between the control tracer and the tracer under validation can then be performed with increasing levels of rigour.

***Qualitative comparison of reconstructed flow structures***

An initial assessment can be performed through qualitative comparison of spatial flow structures derived from the PEPT trajectories. This typically involves visualisation of time-averaged fields such as velocity vectors, occupancy maps, or other derived quantities.

• These visualisations provide an initial verification that the tracer reproduces the expected flow behaviour within the system and identifies the same key flow features as the control tracer. Visualisation may be performed for selected planar slices of the system geometry or by averaging the data along one spatial dimension, depending on the symmetry and nature of the imaged system.

For example - Fig. 16 shows azimut52y averaged velocity vector fields obtained from PEPT data for a stirred tank at two different impeller speeds (300 and 500 rpm), where this Figure allowed qualitative comparison of a conventional IER against novel yttrium-alginate tracers in the related research article [18]. It is evident that the results from both tracers corresponded well, and were both able to identify key features present in the system; two recirculation loops adjacent to and above the impeller region, as well as significant stagnant regions at the bottom of the tank. Azimuthal averaging over the entire system is appropriate in this case due to the approximate axial symmetry of the stirred tank geometry.

***Quantitative comparison of derived flow fields***

Following qualitative verification, more rigorous comparisons can be performed using quantitative statistical analysis of the derived flow fields. Once such approach is the construction of parity plots, in which the values of a given flow metric obtained using the novel tracer are plotted directly against those obtained using the conventional tracer for each spatial location (e.g., voxel or pixel). These comparisons may be performed for velocity magnitude, occupancy probability, shear rate, or other derived quantities. Statistical agreement between the two datasets can then be evaluated using metrics such as:

• the coefficient of determination (R²), and
• the root-mean-squared error (RMSE).

A high coefficient of determination (typically R² > 0.9) together with an unbiased distribution of datapoints around the parity line indicates strong agreement between the tracers. The RMSE can also be normalised by a characteristic velocity or other system-specific scale to provide a meaningful reference for the magnitude of the error between the two datasets.

For example, Fig. 17 presents parity plots comparing the normalised velocity magnitude obtained using yttrium-alginate and IER tracers for the azimut52y averaged velocity fields shown in Fig. 16. For the lower impeller speed, an R² value of 0.936 and an RMSE of 0.0122 (1.2% of the impeller tip speed) were obtained. For the higher impeller speed, the corresponding values were R² = 0.939 and RMSE = 0.0130 (1.3% of the impeller tip speed). These results demonstrate strong statistical agreement between the two tracers.

***Comparison of localisation accuracy and tracking performance***

Finally, the performance of the tracer in terms of localisation accuracy during trajectory reconstruction can be assessed using the error metrics provided by the PEPT processing algorithm. Most PEPT algorithms report a measure of the localisation uncertainty at each timestep based on the spatial distribution of the detected LORs used to determine the particle position. Although the specific form of this metric may vary between algorithms, the validation procedure remains consistent provided that the same algorithm and processing parameters are used for both tracers.

The positional error metric can thus be plotted as a function of time for the duration of the experiment, and can then be used to assess if both tracers show a similar error profile over time (i.e. no tendency for the positional error to increase due to e.g. radioactive leaching, no period of very small constant error due to the tracer becoming localised at one position, etc.). The mean localisation error over the entire dataset may also be calculated to provide a single quantitative metric for comparison, where ideally the novel tracer produces the same or lower overall error.

For example, Fig. 18 shows the variation in positional uncertainty over time for the dataset described in the related research article [18]. In this case the localisation error is defined as the root-mean-squared distance between the minimum-distance point and the set of LORs used to determine the particle position, as produced by the Birmingham method PEPT algorithm. The figure presents both the time-averaged error (calculated over 1-minute intervals) and a boxplot summarising the distribution of localisation errors across the full experiment. These results demonstrate that the positional accuracy obtained using a yttrium-alginate tracer is comparable to that obtained using a conventional IER tracer.

### Assessment of uncertainty and statistical convergence

In addition to direct comparison of reconstructed flow metrics, it is also important to consider the uncertainty associated with PEPT measurements. Sources of uncertainty arise from factors such as detector coincidence statistics, the spatial distribution of the LORs used for particle localisation, tracer activity, and attenuation effects within the system. These factors influence the positional accuracy of the reconstructed trajectory and therefore propagate into the derived quantities such as velocity, occupancy, and shear rate fields which are used for the above validation study.

The localisation error metrics provided by PEPT reconstruction algorithms provide a useful indicator of the instantaneous uncertainty in particle position. When combined with sufficiently long acquisition times, this allows the statistical convergence of time-averaged flow metrics to be assessed. Consistent localisation error distributions and statistically stable flow fields between the control tracer and the tracer under validation provide evidence that the novel tracer does not introduce additional measurement uncertainty relative to conventional PEPT tracers.

## Limitations

The described method for generating versatile alginate-based tracers for nuclear imaging via particle tracking presents several important limitations, which the authors hope to overcome in future publications using novel techniques:

1. **Tracer size:** Hydrogel beads formed through simple extrusion dripping are practically constrained to diameters of at least ∼1 mm, corresponding to xerogels exceeding 400 *μ*m. This limitation arises from the interplay between nozzle diameter, solution viscosity, and pressure drop. While smaller droplets are theoretically achievable by reducing nozzle size, the high viscosities associated with solution 1 impose pressure-drop limits that restrict miniaturisation. Achieving sub-millimetre beads therefore requires more sophisticated methods, such as electro-spraying, microfluidics, or continuous heating of the solution [21,60].
2. **Extrusion dripping throughput:** Maintaining the dripping regime requires low flow rates, and the batch nature of the ExD set-up further reduces production efficiency. Although this is not problematic for discrete particle tracking, where only 1–4 tracers are typically needed, it becomes a significant bottleneck for applications and/or characterisation methods that require larger sample quantities, such as standard particle size distribution measurements via light scattering.
3. **Achievable tracer physico-chemical and radiochemical properties:** The method currently offers limited flexibility in tailoring tracer properties:
  a. *Density:* Hydrogels produced by this approach have densities close to that of water, while xerogels reach an upper limit of ∼2.3 g/cm^3^. Broader density control may be possible in future by encapsulating materials of differing density or by refining drying techniques to preserve hydrogel porosity, both which will require novel or altered bead synthesis and radiolabelling methods. Improved drying methods could also yield more uniform, spherical xerogel particles.
  b. *Radiochemical properties:* Although hydrogels can in principle be radiolabelled using the above low- temperature method, the resulting radiochemical properties are generally poor, limiting the feasibility of low-density tracers. Incorporating high-affinity adsorption agents, such as nanoparticles with strong fluoride affinity, may enhance radiolabelling performance. Methods to incorporate such agents through development of those described herein will be explored in forthcoming studies.

## Conclusion

This work presents a facile and reproducible methodology for the preparation, characterisation, and radiolabelling of customisable alginate-based tracer particles for positron imaging. By combining a simple extrusion-dripping synthesis procedure with the choice of either a low-temperature and evaporative adsorption radiolabelling strategy, the method enables precise control over tracer size, morphology, density, mechanical properties, and radiochemical performance while remaining compatible with stringent positron imaging requirements. The key novelty lies in the ability to tailor key tracer properties through minor formulation or preparation adjustments. This is achieved through the use of a porous hydrogel with high fluoride affinity and the ability to be converted to a smaller-scale xerogel of differing physico-chemical properties. This study also provides the first detailed description of PEPT tracer preparation and radiolabelling methods, addressing a significant gap in the literature and providing ample opportunity for future development and optimisation of particle-based tracers for both PET and PEPT.

## Ethics statements

Not applicable.

## CRediT authorship contribution statement

**Chloe Huckvale Bruno:** Conceptualization, Methodology, visualisation, Data Curation, Investigation, Writing — original draft, Writing — review & editing, Formal analysis. **Daniele Baiocco:** Data Curation, Formal analysis. **William J. Peace:** Investigation, Data Curation, Formal analysis. **Dawid Hampel**: Investigation, Resources. **Manikandan Kadirvel:** Supervision, Resources. **Ben Phoenix**: Resources, Supervision. **Emma Catterson**: Resources, Supervision. **Zhibing Zhang**: Supervision, Project administration. **Carl Wheldon**: Supervision, Project administration, Funding acquisition. **Christopher Windows-Yule:** Writing — review & editing, Supervision, Project administration, Funding acquisition.

## Acknowledgments

The lead author is sponsored by the EPSRC Prosperity Partnership with Aquapak Polymers (Grant number EP/Y025008/1). The authors would like to recognise the valuable contributions of the staff at the University of Birmingham Cyclotron Facility, who supplied the radioactive water, and thank them for their support throughout the experimentation period. Acknowledgement is also given to the Engineering and Physical Sciences (EPS) Workshop at the University of Birmingham for supporting the production of cyclotron target parts and manufacturing spares required to keep experiments on track. Thanks are also given to the Royal Society, who supported the necessary collaborations through the Industry Fellowship of Dr. Windows-Yule (Fellowship number IF\R2\2,320,025).

## Declaration of competing interest

The authors declare that they have no known competing financial interests or personal relationships that could have appeared to influence the work reported in this paper.

## Data Availability

Data will be made available on request.

## Appendix Supplementary materials

Image, application 1

Image, application 2

## Footnotes

IV The elastic limit refers to the fractional deformation at which there is a distinct transition from elastic to plastic (irrecoverable) deformation.

## Footnotes

I A xerogel refers to a dry, somewhat porous structure obtained through the evaporative drying of a wet gel [61].

II Typically taken to be 0.5, since the alginate beads can be assumed to be incompressible at high compression speeds [cxw, gdn].

III The micromanipulation rig is a bespoke piece of equipment based on the diametrical parallel compression of individual particles. It was constructed by the research group of Prof. Zhibing Zhang at the University of Birmingham [53,62], whose operation has been extensively described and validated in numerous research articles [53,[56], [63], [64]]
