# Supplementary material for: Advancing Positron Imaging with Alginate-Based Tracers: Design, Synthesis, and Radiolabelling Methods
Source: MethodsX. 2026 Apr 22;16:103927. doi: 10.1016/j.mex.2026.103927 (PMC13157094; doi:10.1016/j.mex.2026.103927)
Supplement: Supplementary file 2 [file mmc2.docx]

**Supplementary material *and/or* additional information [OPTIONAL]**

#### Example Calculation of Extrusion Dripping Parameters

The parameters in Figure 4 can be selected following guidance from design equations originally outlined by Lindblad and Schneider (1965) [22] and later elaborated by Chan et al. (2009) [23]. This example demonstrates the use of the design equations 1-6 to calculate key extrusion dripping set-up parameters for a specific case, using the parameters specified in Table A1.

To begin, equations 1 – 2 are solved simultaneously:

$$d_{p}=k_{LF}\times0.79\times\left( 6\times\frac{d_{o}\times0.057}{1022\times9.81} \right)^{\frac{1}{3}}$$

$$k_{LF}=0.98-0.04(d_{o}\times1000)$$

$$\Longrightarrow d_{p}=0.0015=(0.98-40d_{o})\times0.79\times\left( d_{o}\times\frac{6\times0.057}{1022\times9.81} \right)^{\frac{1}{3}}$$

$$\Longrightarrow d_{o}=0.00022 \text{m}$$

Thus, a nozzle of approximately 0.2 mm in outer diameter is required. A 33G syringe needle approximately fits this specification, with outer diameter of 0.21 mm and corresponding internal diameter of 0.108 mm. Recalculating the predicted droplet diameter from equation 1 thus gives $d_{p}=0.00148$ mm, only 1.4% off the initial droplet size specification.

Table A1 - Example values used to demonstrate design and set-up of extrusion dripping process.

| Input parameter | Value | Units |
| --- | --- | --- |
| Formulation of gelled beads | A low MGR in solution 1 at 4 wt.%, dripped into an 8 wt.% solution of YCl_3_ salt in distilled water, forming a yttrium-alginate hydrogel bead. | - |
| Solution 1 apparent viscosity | 2.7 | Pa s |
| Solution 1 density | 1022 | kg/m^3^ |
| Solution 1 surface tension | 0.057 | N/m |
| Desired gelled hydrogel bead diameter | 0.0015 | m |
| Known average shrinkage factor | 0.79 | - |

The maximum flowrate can then be determined using equation 3:

| $F_{max}=\frac{\pi d_{i}^{2}}{2}\sqrt{\frac{\gamma}{\rho d_{i}}}=\frac{\pi\left( 0.000108 \right)^{2}}{2}\sqrt{\frac{0.057}{1022\times0.000108}}=1.32\times{10}^{-8} \text{m}^{3}/\text{s}=0.790 \text{ml}\text{/}\text{min}.$ |  |
| --- | --- |

In practice, this denotes the maximum flow rate upper boundary, and a much lower flow rate should be applied to ensure the pump pressure is not excessive and a stable dripping regime that gives reproducible, spherical beads is maintained. Hence, a value of 0.4 ml/min is selected, ensuring stable production and manageable throughput.

The Ohnesorge number is then evaluated from equation 6:

$$\text{Oh}=\frac{\mu}{\sqrt{\rho\frac{d_{p}}{k_{\mathrm{SF}}}\gamma}}=\frac{2.7}{\left( 1022\times\frac{0.00148}{0.79}\times0.057 \right)^{1/2}}=8.17$$

Since the Oh number is > 0.24 and < 11, it is assured that it is physically possible to obtain a uniform and spherical droplet, given the falling distance is within the required ranges. The optimal falling distance within which spherical droplets can be obtained is calculated from equations 4 – 5:

| $D_{min}=1.63e^{0.12\times8.17}=4.35 \text{cm}$ |  |
| --- | --- |
| $D_{max}=62.35\ln\text{(2.97)}+111=242 \text{cm}$ |  |

Thus, the required nozzle dimensions, maximum allowable flowrate, and range for the optimal droplet falling height have now been specified.

#### Calculation Tool for Bead Activity After Radiolabelling

The selection of radiolabelling input parameters can be guided by considering the estimated final tracer bead activity, $A_{b}$, as calculated from equation 10. The spreadsheet named “Supplementary materials - Tracer activity calculation tool.xlsx” has been provided, where the inputs can be altered to observe the change in the final bead activity.
